# Plant functional diversity is affected by weed management through processes of trait convergence and divergence

**DOI:** 10.3389/fpls.2022.993051

**Published:** 2022-10-07

**Authors:** Jose G. Guerra, Félix Cabello, César Fernández-Quintanilla, José M. Peña, José Dorado

**Affiliations:** ^1^ Instituto de Ciencias Agrarias (CSIC), Madrid, Spain; ^2^ Escuela Técnica Superior de Ingeniería Agronómica, Alimentaria y de Biosistemas (ETSIAAB), Universidad Politécnica de Madrid, Madrid, Spain; ^3^ Instituto Madrileño de Investigación y Desarrollo Rural, Agrario y Alimentario (IMIDRA), Finca El Encín, Madrid, Spain

**Keywords:** biodiversity, agricultural management, spontaneous plant cover, mowing, tillage, herbicide, Mediterranean vineyards

## Abstract

Weed management involving tillage and/or herbicides has generally led to a decline of plant diversity in agroecosystems, with negative impacts on ecosystem services provision. The use of plant covers has become the predominant alternative in vineyard management, with numerous studies focusing on analyzing the advantages and disadvantages of plant covers compared to the aforementioned management. Although the impacts of weed management on taxonomic diversity have been widely studied, many gaps remain on their effects on plant functional diversity. As plant functional diversity is linked to the delivery of key ecosystem services in agroecosystems, understanding these effects could enable the development of more sustainable practices. From 2008 to 2018, a long-term trial was carried out in a Mediterranean vineyard to assess different agricultural practices. In this article, we examined how weed management, as well as irrigation use, could affect plant functional diversity. Based on 10 functional traits, such as plant height, specific leaf area or seed mass, we measured different indices of functional diversity and used null models to detect processes of trait convergence and divergence. Our results revealed that weed management and irrigation use had a significant effect on plant functional diversity. Mown plots showed the highest functional richness but were functionally convergent, since mowing was a strong functional filter on most of the traits. Tillage also behaved as a functional filter on some vegetative traits, but favored the divergence of certain reproductive traits. Herbicide-treated and irrigated plots showed the highest values of functional divergence by promoting more competitive species with more divergent trait values. The effect of weed management on these community assembly processes was shaped by the use of irrigation in vineyard rows, leading to functional divergence in those vegetative traits related to resource acquisition and seed mass. These results suggest that greater functional diversity may be associated with the bias caused by higher occurrence of competitive species (e.g. *Convolvulus arvensis*, *Sonchus asper*) with contrasting values for certain traits. Therefore, since these species are considered harmful to crops, higher plant functional diversity might not be a desirable indicator in agroecosystems.

## 1 Introduction

Agricultural intensification is the main process driving the widespread biodiversity loss observed in European farmland in recent decades (Emmerson et al., 2016). This intensification process has caused a marked decline in European arable weeds (Storkey et al., 2012), decreasing both taxonomic and functional diversity (José-María et al., 2010; Carmona et al., 2020), which could have negative impacts on the provision of key ecosystem services in agroecosystems such as crop pollination (Bretagnolle and Gaba, 2015) or pest control (Martínez-Uña et al., 2013; Crowder and Jabbour, 2014). In this context of agricultural intensification, since crop losses due to weeds can be substantial (Oerke, 2006), weed management has targeted weed eradication using recurrent tillage and/or herbicides (MacLaren et al., 2020). These weed management practices have led to environmental impacts such as soil erosion and soil fertility decline, which pose a serious threat to Mediterranean agroecosystems (Novara et al., 2011; Prosdocimi et al., 2016). To deal with these problems, greener weed management practices have been promoted over the past few years. In vineyards, plant cover (usually mown) has become widespread as a viable option to conventional management based on soil tillage, with positive effects such as the improvement of soil properties or the enhancement of biodiversity (Winter et al., 2018; Novara et al., 2019; Guerra et al., 2022), although exhibiting in some cases a reduction on vineyard vigor and yield (Monteiro and Lopes, 2007; Celette and Gary, 2013). The impact of weed management on weed communities has been extensively documented, revealing that management practices such as tillage, herbicide or mowing can exert selective pressure on these communities, altering their composition and/or taxonomic diversity (Doradoand López-Fando, 2006; José‐María et al., 2010; Grundy et al., 2011; Larson et al., 2021; Guerra et al., 2022). However, gaps remain on how these management practices might affect plant functional diversity.

Plant functional diversity can be defined as the value, range, distribution and relative abundance of plant functional traits present in a given ecosystem (Díaz et al., 2007), being closely related to ecosystem properties and provision of key ecosystem services such as carbon sequestration or maintenance of soil fertility (Dıíaz and Cabido, 2001; Díaz et al., 2007; Conti and Díaz, 2013). Thus, plant functional diversity can explain variations in ecosystem functions even when species richness does not (Cadotte et al., 2011). These links to ecosystem processes has made plant functional diversity a central issue for ecology in the last two decades, leading to the proposal of numerous indices for its estimation in different ecosystems (Petchey and Gaston, 2002; Botta-Dukát, 2005; Mason et al., 2005; Villéger et al., 2008).

In agroecosystems, previous works have shown how agricultural intensification reduces functional diversity of weed communities in arable systems (Carmona et al., 2020) and olive groves (Tarifa et al., 2021), although only Carmona et al. (2020) explored in depth the effect on several indicators of functional diversity. However, these studies did not analyze potential differences due to different weed managements. In vineyards, recent short-term studies have partially analyzed the effects of weed management on some aspects of plant functional diversity. Thus, Kazakou et al. (2016) estimated functional richness, one of the three indices proposed by Villéger et al. (2008) to measure functional diversity, finding that spontaneous cover showed higher functional richness than tillage. Hall et al. (2020) estimated functional diversity using Rao’s quadratic entropy (Rao, 1982), observing higher functional diversity in vineyards managed with permanent vegetation cover compared to those observed in bare soil management (herbicide or tillage). Mainardis et al. (2020) found that functional diversity, estimated by functional dispersion and functional divergence, was higher under mowing than under tillage.

Plant functional diversity indices combined with null models have also been used to identify community assembly processes driven by functional traits (Götzenberger et al., 2012; Botta-Dukát and Czúcz, 2016), testing the existence of the two main mechanisms underlying the assemblage of plant communities: habitat filtering and limiting similarity. Trait convergence is usually related to habitat filtering mechanisms (Keddy, 1992), in which environmental or management factors act as abiotic filters restricting the range of species trait values (e.g. Lhotsky et al., 2016). In recent years, it has been described that weed management can affect the functional structure of weed communities favoring one set of functional traits over others (Fried et al., 2012; Guerra et al., 2021) which could be indicative, although not proven, of trait convergence caused by habitat filtering. In contrast, trait divergence could occur according to the principle of limiting similarity (Macarthur and Levins, 1967) which suggests that species can coexist more easily if they diverge in their traits, thereby decreasing competition between them (e.g. Thompson et al., 2010). Nevertheless, convergent and divergent patterns can occur simultaneously within the same community (Bernard-Verdier et al., 2012; Spasojevic and Suding, 2012) and may differ between traits, with convergence being more common for vegetative traits while divergence is more likely for reproductive traits (Grime, 2006). In agroecosystems, the study of these processes that condition plant functional diversity has been limited to grasslands (e.g. Mason et al., 2011; Bernard‐Verdier et al., 2012), lacking studies that test the existence of such processes according to weed management.

The aim of this study was to examine how weed management, as well as irrigation use, might affect functional diversity of weed communities. In particular, we have focused on analyzing the relationship between weed management and trait convergence or divergence patterns. Based on previous studies (Guerra et al., 2021; Guerra et al., 2022), we further hypothesize that functional diversity could also vary depending on the management adjacent to each plot studied. For example, Guerra et al. (2022) reported that species richness was higher in herbicide-treated plots close to mowing than in plots close to tillage. In addition, we have recently observed that at herbicide-treated and irrigated rows, with intermediate values of bare soil, the most competitive species were favored (Guerra et al., 2021; 2022). Given that competition is a factor that can shape plant functional diversity (Navas and Violle, 2009), this work also studied the relationship between functional diversity and competitiveness (CSR strategy; Grime, 1974). Therefore, the purpose of this article is to contribute to increase the knowledge on the influence of agronomic management on plant functional diversity, so that implications for agroecological weed management can be analyzed.

## 2 Materials and methods

### 2.1 Study site and experimental design

Research was carried out in an experimental vineyard at the IMIDRA farm El Socorro (Colmenar de Oreja, Madrid), located at 755 m in the central plateau of the Iberian Peninsula, developed on a clay-loam soil of type Calcic Haploxeralf. Climate is Mediterranean, with mean annual temperature of 13.7°C and annual precipitation of 421.4 mm (data from the farm weather station for the period 2001–2021). Grapevines (c.v. Tempranillo) were grown on trellises, with deficit drip irrigation applied from June to September in vineyard rows.

**Figure 1:**
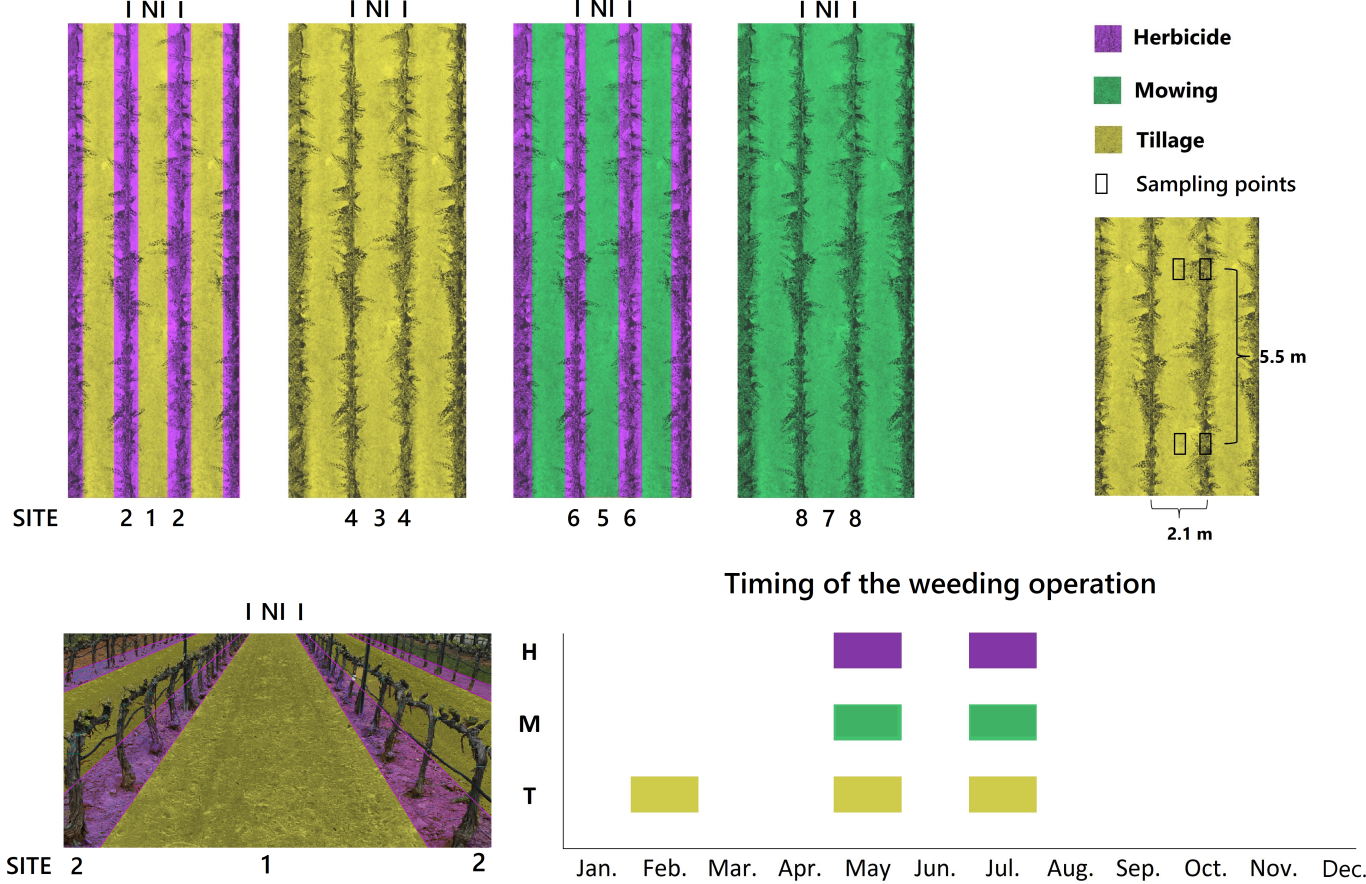
Diagram of the experimental design and location of the sampling points (upper part), 3D image depicting sites 2-1-2 (lower left) and schematic chronogram of the weeding operations carried out in the study field (lower right). Irrigation treatment: I: irrigated row; NI, non-irrigated inter-row. Site: 1, tilled inter-rows adjacent to herbicide-treated plots; 2, herbicide-treated rows adjacent to tilled plots; 3, tilled inter-rows adjacent to tilled plots; 4, tilled rows; 5, mown inter-rows adjacent to herbicide-treated plots; 6, herbicide-treated rows adjacent to mown plots; 7, mown inter-rows adjacent to mown plots; 8, mown rows.

In 2008, a randomized block trial with four replications was set up to assess different vineyard management systems. Based on this trial, a study was designed in 2015 to examine how different weed management (herbicide, tillage, mowing), as a function of irrigation within the vineyard (non-irrigated inter-rows, irrigated rows) could affect the functional diversity of plant communities (**Figure 1**). Irrigation was considered since the deficit irrigation applied during the dry period generates clearly contrasting conditions between inter-rows (non-irrigated) and rows (irrigated) that could have a significant effect on plant functional diversity. Accordingly, five types of plots were identified: a) herbicide-treated rows (irrigated); b) mown inter-rows (non-irrigated); c) mown rows (irrigated); d) tilled inter-rows (non-irrigated); e) tilled rows (irrigated). Herbicide-treated inter-rows (non-irrigated) were not included in the original design as this is a very unusual praxis in Mediterranean vineyards, where herbicide application is restricted to rows. Herbicide treatment consisted of two applications of glyphosate (1440 g ai ha^-1^) throughout the grapevine growth cycle (**Figure 1**). Mowing of the spontaneous plant cover (two passes) was performed with a flail mower in vineyard inter-rows and with an inter-vine mower in rows. Tillage (two-three passes) was carried out with a cultivator in vineyard inter-rows and with an inter-vine tiller in vineyard rows.

According to our initial hypothesis that the functional diversity of a plot could vary as a function of adjacent management, we distinguished eight different environmental sites in the experimental design (**Figure 1**). For example, it was considered that the functional diversity in herbicide-treated rows next to mowing (site 6) might be different from that found in herbicide-treated rows next to tillage (site 2). For further details on study site and experimental design, see Guerra et al. (2022).

### 2.2 Vegetation sampling

In each of the 32 plots (4 blocks × 8 sites), six sampling points separated by a distance of 5.5 m were fixed (**Figure 1**). Sampling points were placed in pairs (inter-row and row, respectively) spaced 1 m apart. In each of them, a 33 × 66 cm frame was placed, identifying all plant species and assessing the coverage percent of each species (Andújar et al., 2010), as well as the percentage of bare soil. For each species, mean percentage cover was calculated by pooling the data obtained in the six sampling points of each plot. Castroviejo (1986-2012) was used for species identification, updating the species name according to the International Plant Names Index (IPNI, 2022).

### 2.3 Plant traits

In order to measure functional diversity, 10 functional traits previously associated with plant response to weed management (Guerra et al., 2021), were selected: five vegetative traits related to resource-acquiring capacity (Raunkiaer life form [RLF], plant height vegetative [PHV], specific leaf area [SLA], leaf dry matter content [LDMC], leaf area [LA]), and five reproductive traits related to regenerative ability (seed mass [SM], seed bank longevity index [SLI], onset of flowering [OFL], duration of flowering period [DFP], dispersal syndrome [DS]). An overview of the traits used can be found in **Table 1**.

**Table 1:** Brief description of the functional traits used tocompute the functional diversity indexes.

Code	Name	Units	Explanation
RLF	Raunkiaer life form	–	The life form of a plant defined by the position and degree of protection of its perennating bud (Raunkiaer, 1934). Qualitative trait with four levels: therophyte, therophyte/hemicryptophyte, hemicryptophyte, geophyte.
PHV	Plant height (vegetative)	cm	The shortest distance between the upper-boundary of the main photosynthetic tissues (excluding inflorescences) on a plant and the ground level (Cornelissen et al., 2003).
SLA	Specific leaf area	mm^2^ mg^-1^	The one-sided area of a fresh leaf divided by its oven-dry mass (Cornelissen et al., 2003).
LDMC	Leaf dry matter content	mg g^-1^	The oven-dry mass (mg) of a leaf divided by its water-saturated fresh mass (g) (Cornelissen et al., 2003).
LA	Leaf area	mm^2^	Area of a leaf.
SM	Seed mass	mg	The oven-dry mass of an average seed of a species (Cornelissen et al., 2003).
SLI	Seed bank longevity index	–	The ratio of the number of records that classify the species as persistent to the number of all records for the species (Thompson et al., 1998). SLI can take any value from 0, when all records are transient, to 1, when all records are persistent.
OFL	Onset of flowering	–	Species classification according to month of beginning of flowering period: earlier (January, February), medium (March, April), late (May, June).
DFP	Duration of flowering period	months	Months during which the flowering occurs.
DS	Dispersal syndrome	–	Species grouped according to their dispersal mechanisms into three categories: unspecialized, refers to autochorous species or species without any specific dispersal mechanism; anemochorous, dispersed by wind; zoochorous, dispersed by animals.

This study included only species with a significant presence, excluding in the analyses those with less than 0.25% relative cover based on the overall value of all plots. Then, plant trait data were compiled from plant trait databases such as TRY Database (Kattge et al., 2020) and stored in a new database (Guerra et al., 2021). Further details on the traits and database used can be found in Guerra et al. (2021).

### 2.4 Functional diversity analysis

#### 2.4.1 Functional diversity estimation

Functional diversity based on three components, i.e. functional richness, functional evenness and functional divergence (Mason et al., 2005), were analyzed in this work. Functional richness and functional evenness were computed according to two indices proposed by Villéger et al. (2008): FRic, which quantifies the functional space volume filled by the community based on the calculation of the convex hull volume (see Cornwell et al., 2006); and FEve, which measures the uniformity of abundance distribution in this volume to quantify the regularity with which the different species, weighted by their abundance, fill this functional space.

Functional divergence was measured from Rao’s quadratic entropy (RaoQ), which is often used as an index of functional divergence (Schleuter et al., 2010) although encompasses both richness and functional divergence (Mouchet et al., 2010). Hence, RaoQ is the mean distance between species weighted by species abundance (Botta-Dukát, 2005). This index is strongly related to functional dispersion index (FDis) proposed by Laliberté and Legendre (2010) and to the community weighted variance (CWV) defined by Sonnier et al. (2010). It should be noted that FDis and CWV were initially calculated, yielding very similar results to RaoQ (**Supplementary Figure 1**). ConsequentlRaoQ was used because it is a more common index than the previous ones in plant functional diversity studies.

For computing functional diversity indices, a functional distance matrix based on trait values for each species was initially generated using the Gower distance (Gower, 1971). On this functional matrix, a principal coordinate analysis (PCoA) was performed and the resulting PCoA axes were used as new “traits” to compute in each plot the indices mentioned above, using the function *dbFD* from the R package *FD* (Laliberté and Legendre, 2010).

#### 2.4.2 Null models: Estimating effect size FRic and effect size RaoQ

Thereafter, a null model approach was applied for functional diversity indices (except for FEve, which was excluded according to Mason et al., 2013), testing whether observed FRic and RaoQ values, the latter calculated for the set of traits and also for each trait individually, differed from random expected values. This approach was implemented for two purposes: Firstly, to remove any trivial effect of species richness on functional diversity indices (in line with Mason et al., 2013; Pakeman et al., 2017; Carmona et al., 2020). This is particularly relevant for FRic, given its strong positive correlation with species richness (Villéger et al., 2008; Mouchet et al., 2010) such as was revealed in a preliminary analysis (Supplementary **Figure 2**). After removing this effect, RaoQ becomes a “pure measure” of functional divergence (Mason et al., 2013). Secondly, null models were developed to test changes in community assembly processes (similarly to Bernard‐Verdier et al., 2012; Mason et al., 2012; Mason et al., 2013). Although both RaoQ and FRic have proven to be reliable measures for this purpose, RaoQ seems to be the most suitable for detecting trait convergence and divergence (Botta-Dukát and Czúcz, 2016).

**Figure 2:**
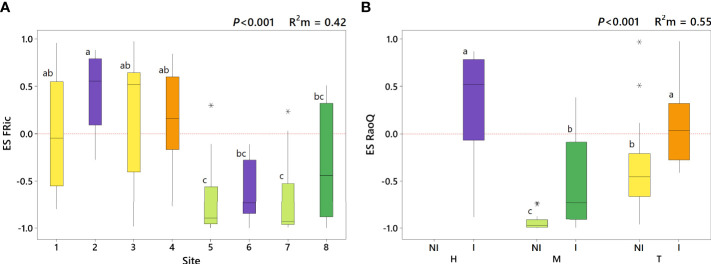
Effect size of FRic (ES FRic) depending on site **(A)** and effect size of RaoQ (ES RaoQ) depending on the interaction of weed management and irrigation (WM:I) **(B)**. Marginal *R* ^2^ (*R* ^2^m) provides the variance explained by the fixed effects. Same letters on the boxes indicate no significant differences. H, herbicide; M, mowing; T, tillage; NI, non-irrigation; I, irrigation. Sites: 1, tilled inter-rows adjacent to herbicide-treated plots; 2, herbicide-treated rows adjacent to tilled plots; 3, tilled inter-rows adjacent to tilled plots; 4, tilled rows; 5, mown inter-rows adjacent to herbicide-treated plots; 6, herbicide-treated rows adjacent to mown plots; 7, mown inter-rows adjacent to mown plots; 8, mown rows. Outliers are indicated with an asterisk (*).

Implemented null models were selected based on previous work (Mason et al., 2013; Botta-Dukát and Czúcz, 2016; Götzenberger et al., 2016). For FRic, randomized matrix was obtained with the independent swap algorithm (function “independentswap” in *picante*) while maintaining the occurrence frequency of the species and the species richness of each plot. For RaoQ, species abundance within each plot was shuffling maintaining sample species richness (function “richness” in *picante*). Null models were generated from randomized matrices using the R package *picante* (Kembel et al., 2010), performing 1000 randomizations for each model to ensure an accurate estimate (Gotelli, 2000).

Subsequently, functional diversity indices were calculated from randomized matrices, measuring the degree to which the observed values of a variable were different from its expected values and later calculating the effect size. The standard method for measuring effect size in a null model is to compute standardized effect size (SES; Gotelli and McCabe, 2002), but Botta-Dukát (2018) has recently pointed out that this method is not suitable when, as in our case, the symmetry assumption is not satisfied. For this reason, it was decided to measure the effect size (ES) according to Chase et al. (2011) as has been performed previously for other cases with not-symmetric distribution of the null model (e.g. Bernard‐Verdier et al., 2012; Lhotsky et al., 2016):


ES=(p − 0.5)×2

where


$$p=\;\frac{number\;\left(NULL<obs\right)+\frac{number\;\left(\;NULL=obs\right)}{2}}{1000}$$

being *number (NULL< obs)* the number of occasions in which the value obtained from the null model (*NULL*, expected value) was less than the observed value (*obs*) and *number (NULL = obs)* the number of occasions in which the value obtained from the null model was equal to the observed value. This procedure was performed separately for both FRic and RaoQ. Thus, two new indices were obtained from the effect sizes of FRic (ES FRic) and RaoQ (ES RaoQ) with values ranging from +1 to -1, where positive values indicate a higher observed value than expected, and vice versa. Trait convergence or divergence were assumed if the observed RaoQ values were, respectively, lower or higher than the estimated RaoQ values by the null model (similarly to Bernard‐Verdier et al., 2012; Boonman et al., 2021). Since sometimes community assemblage patterns cannot be described by a single multivariate trait index (Spasojevic and Suding, 2012; Carmona et al., 2015), ES RaoQ has been calculated for the set of all traits (multi-trait) and for each single trait. Besides, although RaoQ (and by extension, ES RaoQ) is less affected by trait outliers than FRic (Cornwell and Ackerly, 2009), functional divergence will be higher the closer the abundant species are located to the boundary of the functional trait space (Botta-Dukát and Czúcz, 2016). Taking this into account, it was assumed that the occurrence of abundant species with extreme values for certain traits could be associated with trait divergence. Abundant species were considered to be those dominant and subordinate species (Grime, 1998) with a relative cover of more than 3% (arbitrary value), which could fundamentally govern the community assemblage processes under the mass ratio hypothesis (Grime, 1998; Dıíaz and Cabido, 2001). Therefore, the relationship between the most abundant species and ES RaoQ was also analyzed.

### 2.5 Competitiveness index

The CSR theory (Grime, 1974), which has been extensively applied to the study of plant communities (e.g. Cerabolini et al., 2010; Guerra et al., 2021), categorizes plant species within a triangular scheme consisting of three dimensions: a competitiveness dimension (C-dimension), a stress-tolerance dimension (S-dimension) and a ruderality dimension (R-dimension). The competitive dimension is related to the ability of plant species to compete with their neighbors, with competitor plants typically being large herbaceous species with rapid resource acquisition (Grime, 1974; Hodgson et al., 1999). Building on this theoretical framework, Hodgson et al. (1999) developed a method for CSR classification based on functional traits of herbaceous plants.

Recently, a correlation between the functional structure of weed communities and the CSR strategy has been observed (Guerra et al., 2021). In order to analyze the relationship between competitiveness and functional diversity, we applied a procedure similar to that used by Ricotta et al. (2016) to calculate here a competitiveness index (C_index_). For this purpose, the competitiveness dimension (C-dimension) of each of the selected species was firstly estimated according to Hodgson et al. (1999). Then, community-weighted mean (CWM; Garnier et al., 2004) of C-dimension values were computed with the R package *FD* for each of the plots (i.e. plot-level C-dimension values weighted by species abundance). Thus, C_index_ was calculated as:


$$C_{\text{index}}=\sum_{\text{i}=1}^{\text{n}}p_{i}\times {\text{C‐dimension}}_{i}$$

where *p_i_* is the relative abundance of species *i*, and C-dimension*_i_* is the C-dimension value of species *i*.

### 2.6 Statistical analyses

The effect of weed management (WM) and irrigation (I) on functional diversity indices was analyzed by means of linear mixed models with the R package *lme4* (Bates et al., 2015), except for RaoQ models which were fitted *via* beta regressions models, with “loglog” as link function, using the R package *betareg* (Cribari-Neto and Zeileis, 2010). Due to the inherent constraints of the experimental design, WM and I were always considered as nested fixed factors (WM:I), although the individual effects of WM and I were also explored. In addition, the influence that adjacent management might have on each plot was examined by fitting alternative models with “site” as a fixed effect. In all fitted models, year and block were considered as random effects.

To screen the best models for each variable, the corrected Akaike’s information criterion (AICc) was used, choosing the model with the lowest AICc value (Burnham and Anderson, 2002). Model fit was estimated by mean of pseudo R^2^ (only for beta regression models), marginal R^2^ (variance explained by fixed effects) and conditional R^2^ (variance explained by the whole model) using the R package *performance* (Lüdecke et al., 2021). To test whether fixed effects of the selected models were statistically significant, type-III ANOVA tests were performed using the R package *car* (Fox and Weisberg, 2019). When a significant effect was observed, a *post-hoc* analysis was performed using the pairwise t-test adjusted for Bonferroni correction for pairwise comparison.

In order to detect trait convergence or divergence patterns, values of ES RaoQ were tested as to whether they were significantly different from zero using a one-sample t-test (for normally distributed data) and a Wilcoxon signed-ranks test (for non-normally distributed data). In addition, to analyse the impact of the most abundant species on trait divergence, the relationships between ES RaoQ (both multi-trait and single-trait estimations) and relative cover of these species were assessed using Pearson’s correlation test (when data were fitted to a normal distribution) and Spearman’s correlation test (for non-normally distributed data).

Finally, the relationship between C_index_ and ES RaoQ was explored through linear regression models using ES RaoQ as the response variable. All analyses were carried out in R 4.1.0 (R Core Team, 2022).

## 3 Results

A total of 59 plant species were detected, of which 29 showed a significant occurrence (**Supplementary Table 1**). Complete trait information for these 29 species, reported elsewhere (Guerra et al., 2021), was employed to compute functional diversity indices. Nonetheless, trait data for the most abundant species are provided (see **Supplementary Tables 2**, **3**). All models except the one constructed for ES FRic showed lower AICc values when WM:I was used as predictor variable (**Table 2**).

**Table 2:** Summary of models built to analyze the effect of weed management (WM) and irrigation (I) on functional diversity indices.

	FRic				FEve				RaoQ				ES FRic				ES RaoQ			
	WM	I	WM:I	Site	WM	I	WM:I	Site	WM	I	WM:I	Site	WM	I	WM:I	Site	WM	I	WM:I	Site
*P*-value	**	*ns*	**	**	*	*ns*	*	*	***	***	***	***	***	*ns*	***	***	***	***	***	***
$R_{c}^{2}$	0.17	0.09	0.20	0.26	0.10	0.03	0.11	0.18	-	-	-	-	-	-	-	-	0.49	0.33	0.60	0.62
$R_{m}^{2}$	0.12	0.00	0.16	0.16	0.08	0.03	0.10	0.16	-	-	-	-	0.26	0.03	0.29	0.42	0.45	0.30	0.55	0.56
pseudo *R* ^2^	-	-	-	-	-	-	-	-	0.33	0.26	0.43	0.43	-	-	-	-	-	-	-	-
AICc	-	-	-31.91	-14.81	-	-	-70.98	-61.07	-	-	-525.72	-487.97	-	-	169.89	162.07	-	-	119.83	127.35

FRic, functional richness; Feve, functional evenness; RaoQ, Rao’s quadratic entropy; ES FRic, effect size functional richness; ES RaoQ, effect size Rao’s quadratic entropy. The significance levels of the fixed terms were assessed from a type-III ANOVA (*ns*, non-significant, *P < 0.05, **P < 0.01, ***P < 0.001). For mixed models, $R_{c}^{2}$ and $R_{m}^{2}$ provide the proportion of variance explained by the whole model and by the fixed effects, respectively. Pseudo *R* ^2^ was estimated for beta regression models.

### 3.1 Effects of weed management and irrigation on functional diversity

A moderate influence of WM:I on functional richness ( $R_{m}^{2}$ = 0.16) was observed (**Table 2**). Thus, functional richness was significantly higher in mown irrigated plots compared to tilled irrigated plots, while intermediate values were reached in herbicide-treated irrigated plots (**Table 3**). However, when the influence of species richness was removed (i.e. ES FRic), opposite values were obtained. Model for ES FRic was best fitted when “site” was used as predictor variable ( $R_{m}^{2}$ = 0.44). Thus, herbicide-treated sites (site 2 and 6) showed significantly different ES FRic values between them (**Figure 2A**). Indeed, herbicide-treated rows next to tillage (i.e. site 2), showed similar values to tilled plots but significantly greater than herbicide-treated rows next to mowing (i.e. site 6). A slightly lower effect of WM:I on functional evenness was found ( $R_{m}^{2}$ = 0.10), with no significant differences observed among managements. In contrast, a marked influence of WM:I on functional divergence (RaoQ) was observed (pseudo *R* ^2^ = 0.43), reaching significantly greater values in herbicide-treated irrigated plots than in mown irrigated plots (**Table 3**). This effect is even more marked for ES RaoQ ( $R_{m}^{2}$ = 0.55). Besides, irrigation also had a significant effect on functional divergence (**Table 2**). Thus, higher RaoQ and ES RaoQ values were observed in irrigated plots, although for RaoQ, these differences were only significant when comparing among mown plots (**Table 3**).

**Table 3:** Mean values (± SE) by plots of functional diversity indices based on the interaction of weed management and irrigation (WM:I).

	FRic	FEve	RaoQ	ES FRic	ES RaoQ
**HI**	0.41 ± 0.03 **ab**	0.58 ± 0.03 **a**	0.06 ± 0.00 **a**	-0.05 ± 0.15 **a**	0.31 ± 0.13 **a**
**MNI**	0.45 ± 0.03 **ab**	0.49 ± 0.02 **a**	0.03 ± 0.00 **c**	-0.71 ± 0.08 **b**	-0.94 ± 0.01 **c**
**MI**	0.54 ± 0.04 **a**	0.52 ± 0.04 **a**	0.04 ± 0.00 **b**	-0.41 ± 0.17 **ab**	-0.50 ± 0.15 **b**
**TNI**	0.36 ± 0.05 **b**	0.56 ± 0.03 **a**	0.04 ± 0.00 **b**	0.12 ± 0.12 **a**	-0.38 ± 0.09 **b**
**TI**	0.29 ± 0.04 **b**	0.61 ± 0.04 **a**	0.06 ± 0.00 **ab**	0.17 ± 0.14 **a**	0.10 ± 0.13 **a**

HI, herbicide – irrigation; MNI, mowing – non-irrigation; MI, mowing – irrigation; TNI, tillage – non-irrigation; TI, tillage – irrigation.

FRic, functional richness; Feve, functional evenness; RaoQ, Rao’s quadratic entropy; ES FRic, effect size of functional richness; ES RaoQ, effect size of Rao’s quadratic entropy. Means within the same column with the same letters are not significantly different.

### 3.2 Trait convergence and divergence as a function of weed management and irrigation

**Figure 3:**
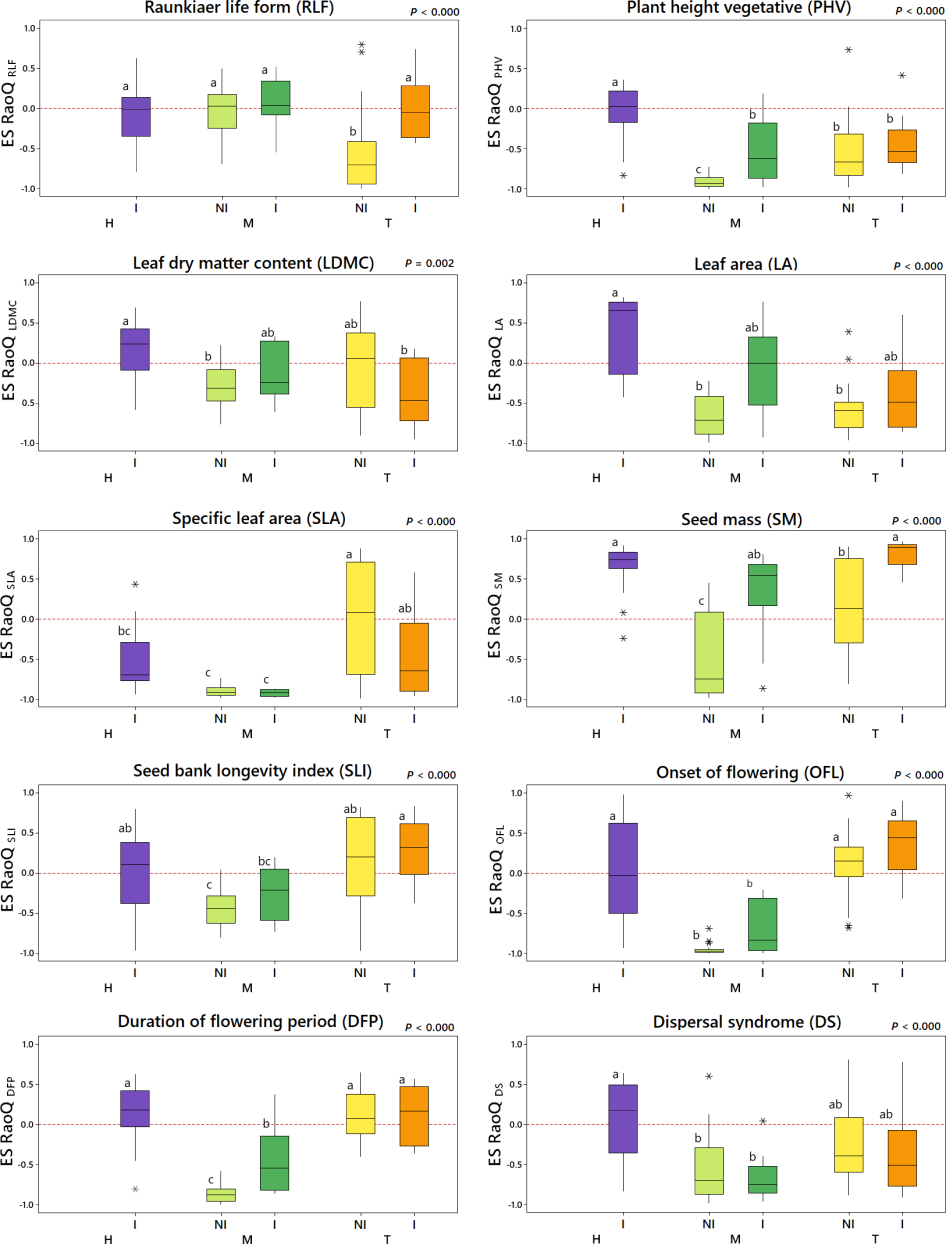
Effect size of RaoQ (ES RaoQ) for each single trait depending on the interaction of weed management and irrigation (WM:I). H, herbicide; M, mowing; T, tillage; NI, non-irrigation; I, irrigation. For pairwise comparison, a Student’s t-test was conducted, with *P*-values adjusted using the Bonferroni correction. Same letters on the boxes indicate no significant differences. Trait code indicated in the subscripts: RLF, Raunkiær Life Form; PHV, plant height; LDMC, leaf dry matter content; LA, leaf area; SLA, specific leaf area; SM, seed mass; SLI, seed bank longevity index; OFL, onset of flowering; DFP, duration of flowering period; DS, dispersal syndrome. Outliers are indicated with an asterisk (*).

Results related to ES RaoQ for the whole set of traits showed a process of trait convergence in tilled non-irrigated plots and in mown plots, being especially marked in mown non-irrigated plots, while trait divergence was observed in herbicide-treated irrigated plots (**Figure 2B**). Therefore, mowing significantly promoted trait convergence while trait divergence was enhanced in irrigated plots, supporting what was observed in the exploratory analysis (**Supplementary Tables 4**, **5**). The results of ES RaoQ values for each single trait provided more detailed information (**Figure 3**; **Table 4**). Trait convergence observed in tilled non-irrigated plots was mainly due to the negative ES RaoQ values detected for RLF, PHV and LA. In mown plots, convergence was found for all traits except for RLF and, in the case of mown irrigated plots, also for LA and SM. In contrast, a trend towards trait divergence was observed in most traits in herbicide-treated irrigated plots, but this divergence was only significant for LA and SM. It should be noted that a strong functional convergence was observed for PHV in all plots except in herbicide-treated irrigated plots, where the median value was above zero. In tilled irrigated plots, high functional divergence was also observed for SM and, to a lesser extent, for SLI and OFL. However, the ES RaoQ value for the whole set of traits was not significantly higher than zero, since trait convergence was detected for leaf traits and PHV. In addition, when the effect of irrigation on the divergence of single traits was explored, it was revealed that irrigation only had a significant effect on divergence for RLF, PHV, LA and SM (**Supplementary Table 5**). Thus, for these traits, irrigated plots were more functionally divergent than non-irrigated plots.

**Table 4:** Results of tests on ES RaoQ values to analyze trait convergence or divergence, using a one-sample t-test (for normally distributed data) and a Wilcoxon signed-rank test (for non-normally distributed data) to test whether ES RaoQ values are significantly different from 0.

	ES RaoQ	ES RaoQ _RLF_	ES RaoQ _PHV_	ES RaoQ _LDMC_	ES RaoQ _LA_	ES RaoQ _SLA_	ES RaoQ _SM_	ES RaoQ _SLI_	ES RaoQ _OFL_	ES RaoQ _DFP_	ES RaoQ _DS_
HI	0,52*	-0,08	*0,02*	0,16	0,66**	-0,70***	0,74***	0,01	*-0,03*	*0,18*	*0,18*
MNI	-0,97***	0,03	-0,93***	-0,29***	-0,71***	-0,91***	-0,74***	-0,43***	-0,98***	-0,88***	-0,69***
MI	-0,73**	0,10	-0,54***	-0,12	-0,08	-0,92***	*0,55*	-0,25*	-0,83***	-0,43**	-0,75***
TNI	-0,46**	-0,70***	-0,67***	-0,06	-0,59***	*0,08*	*0,13*	*0,20*	0,16	0,09	-0,39*
TI	0,01	0,02	-0,43**	-0,37**	-0,40**	-0,41*	0,81***	0,29*	0,44**	0,14	-0,34

HI, herbicide – irrigation; MNI, mowing – non-irrigation; MI, mowing – irrigation; TNI, tillage – non-irrigation; TI, tillage – irrigation. Trait code indicated in the subscripts: RLF, Raunkiær Life Form; PHV, plant height; LDMC, leaf dry matter content; LA, leaf area; SLA, specific leaf area; SM, seed mass; SLI, seed bank longevity index; OFL, onset of flowering; DFP, duration of flowering period; DS, dispersal syndrome. For normally distributed data, mean values are indicated. For non-normally distributed data, median values are reported (in italics). Significance levels are indicated by *P*-values: *P < 0.05; **P < 0.01; ***P < 0.001

**Figure 4:**
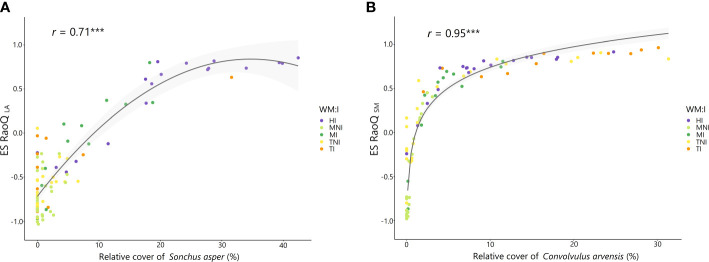
Relationship between the relative cover of *Sonchus asper* and effect size of RaoQ for leaf area (ES RaoQ _LA_) **(A)** and between the relative cover of *Convolvulus arvensis* and effect size of RaoQ for seed mass (ES RaoQ _SM_) **(B)**. Dots, corresponding to plots, have been filled in by the interaction of weed management and irrigation (WM:I): HI, herbicide – irrigation; MNI, mowing – non-irrigation; MI, mowing – irrigation; TNI, tillage – non-irrigation; TI, tillage – irrigation. For each correlation, Spearman correlation coefficient (*r*) and its level of significance (^***^ *P*< 0.000) are shown. Confidence intervals (95%) are shaded in light gray.

The highest ES RaoQ values found for PHV, LA and SM were strongly associated with two species having more extreme values for these traits: *Sonchus asper* (for PHV and LA) and *Convolvulus arvensis* (for SM) (**Supplementary Figure 4**). An increased occurrence of *S. asper* caused a substantial increase in ES RaoQ for PHV and especially for LA, with ES RaoQ values above 0.5 for plots with a relative cover above 20% for this species (**Figure 4A**); while a small increase in the percentage of *C. arvensis* caused an exponential increase in ES RaoQ for SM (**Figure 4B**), with ES RaoQ higher than 0.5 from a percentage of *C. arvensis* of 4%. For further details, see **Supplementary Figures 5**, **6**.

### 3.3 Links between competitiveness and functional diversity

**Figure 5:**
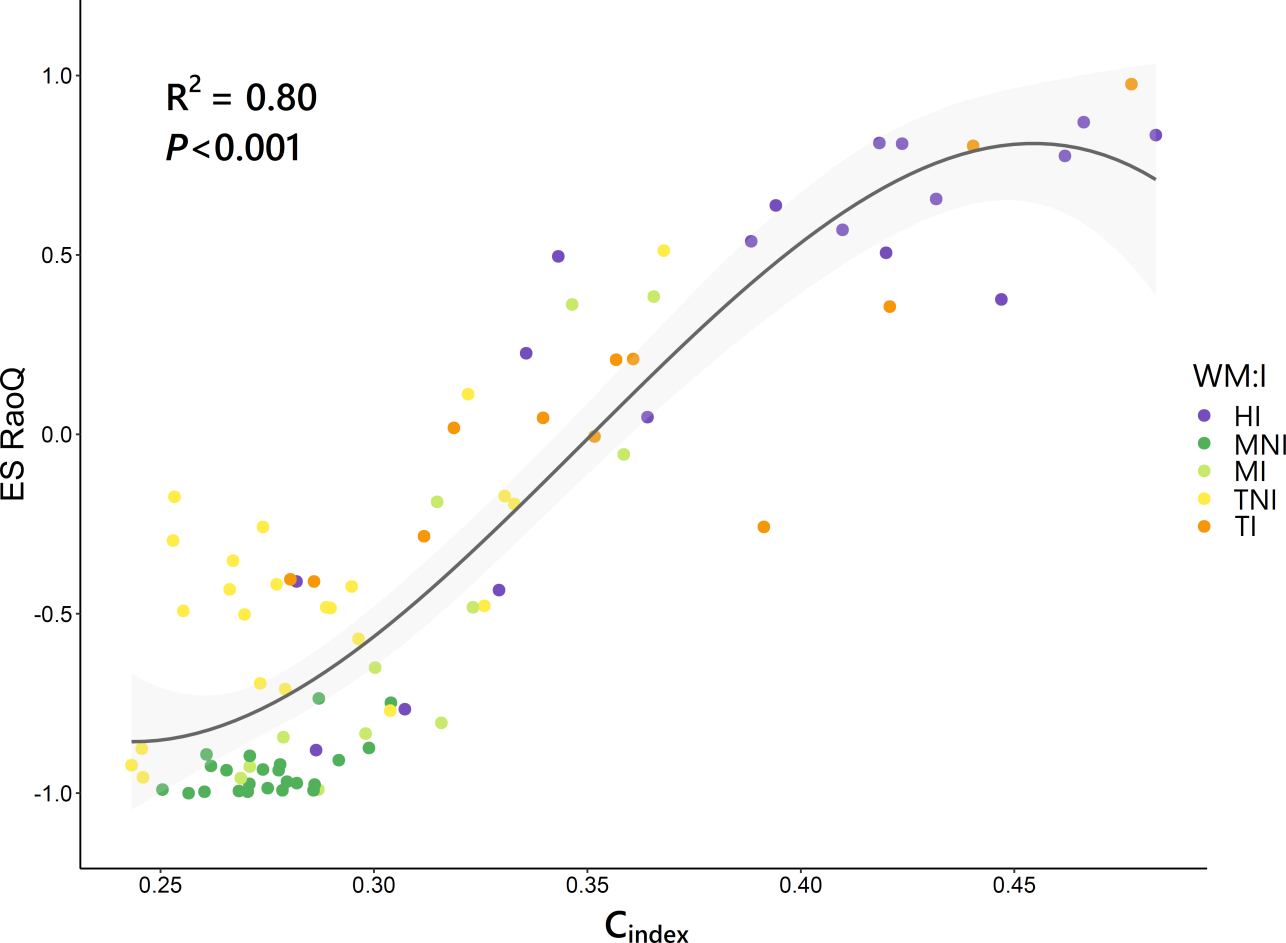
Relationship between competitiveness index (C_index_) and the effect size of RaoQ (ES RaoQ). Dots, corresponding to plots, have been filled in by the interaction of weed management and irrigation (WM:I): HI, herbicide – irrigation; MNI, mowing – non-irrigation; MI, mowing – irrigation; TNI, tillage – non-irrigation; TI, tillage – irrigation. R-squared (*R* ^2^) provides the proportion of variance explained by C_index_. Confidence intervals (95%) are shaded in light gray.

A strong correlation was found between C_index_ and ES RaoQ (*R* ^2^ = 0.80, *P*< 0.001), which was best fitted using a cubic regression model (**Figure 5**). Hence, functional divergence was greater the higher the competitiveness, but with a slight drop for C_index_ above 0.45. An analysis of the ES RaoQ values for each single trait showed how there was a significant positive correlation between ES RaoQ and competitiveness for all traits except for LDMC and SLA (**Supplementary Table 6**). This correlation was particularly strong for PHV, LA and SM.

## 4 Discussion

To our knowledge, this is the first long-term study addressing how plant functional diversity is shaped by weed management and the first work to evidence trait convergence and divergence processes linked to weed management. Our results revealed that weed management affected different dimensions of functional diversity, with significant major impact on functional divergence. Indeed, trait divergence was observed in herbicide-treated irrigated plots, while trait convergence patterns were detected in tilled plots and especially in mown plots. Irrigation use, which implies different ecological conditions within the vineyard, also affected functional diversity with higher functional divergence in irrigated plots with respect to non-irrigated plots.

### 4.1 Effects of weed management and irrigation on plant functional diversity

The findings obtained for functional richness were within expectations since, as the literature states, a significant positive correlation exists between functional and species richness (Villéger et al., 2008). In our study, mown plots with higher species richness (Guerra et al., 2022) were also those that filled a larger functional space, which is in consonance with the findings of Kazakou et al. (2016). However, when the effect of species richness on functional richness was removed (i.e. ES FRic), the results were reversed. For instance, in mown inter-rows the observed functional richness values were much lower than expected from the null model (ES FRic = -0.71), evidencing high functional redundancy, thus revealing the occurrence in these plots of a large number of species with similar traits. Herbicide effect on ES FRic differed according to adjacent management (**Figure 2A**), similar to that observed for species richness in Guerra et al. (2022). In this previous work it was noted that herbicide-treated plots next to mowing (site 6) showed higher number of species than herbicide-treated plots next to tillage (site 2). Taking this into account, the low ES FRic values observed at site 6 highlight that higher species richness did not lead in this case to a significant increase in functional richness (**Supplementary Figure 3**), suggesting that added species are functionally redundant.

Likewise, functional evenness was also lower in mown plots. These findings are in line with Carmona et al. (2020), who observed the lowest values of functional evenness and SES FRic (very similar to ES FRic) in less intensively managed plots. In addition, the lower values observed for ES FRic and FEve in mown plots suggest the existence of habitat filtering mechanisms (Mouchet et al., 2010; Pakeman, 2011), which is in line with the lower values of ES RaoQ found in these plots. Hence, mowing seems to be acting as an abiotic filter that promotes a process of functional convergence in the vast majority of traits, in line with previous work in meadows (Mudrák et al., 2016; Halassy et al., 2019). These results are in agreement with those discussed by Guerra et al. (2021), who observed a distinct suite of plant traits in response to mowing (species with higher stress tolerance, lower vegetative height, lower SLA and higher LDMC). Nonetheless, our findings are not consistent with previous studies reporting the highest values of RaoQ (or similar index FDis) in spontaneous plant covers (Hall et al., 2020) or mown rows (Mainardis et al., 2020). This could be explained by the fact that mowing effects on plant communities depend on the time scale considered, as we will discuss below. Hence, plant communities tend to become more homogeneous and functionally convergent after years of adopting conservation agriculture practices (Derrouch et al., 2021). In particular, mowing can lead over time to a homogeneous species composition (Lepš, 2014) and to functional convergence in some traits such as plant height (Halassy et al., 2019).

In tilled inter-rows, although in a more attenuated form, it was also possible to detect a process of functional convergence in some vegetative traits (RLF, PHV, LA), due to the abundant occurrence of short ruderal therophytes with small leaves such as *Lamium amplexicaule*, which are favored by tillage (Hall et al., 2020; Guerra et al., 2021). However, contrary to expectations, no trait convergence was observed in tilled non-irrigated plots for leaf economics traits (LDMC, SLA), which had been previously associated with tillage and agricultural intensification (Carmona et al., 2020; Hall et al., 2020; Guerra et al., 2021). Although tillage may be acting as a functional filter enhancing traits related to fast-growing ruderal species (Guerra et al., 2021), this habitat filtering process may have been hidden in tilled non-irrigated plots by two independent processes. Firstly, the high abundance of *Stellaria media* in these plots, with an extreme value for SLA, led to an increase in functional divergence (**Supplementary Figures 5D** and **6D**). High SLA values are associated with high growth rates (Hunt and Cornelissen, 1997), enabling species to complete their life cycle in a short time (Grime and Hunt, 1975). This would give *S. media* an adaptive advantage to proliferate in environments subject to recurrent disturbances, such as tilled fields (Jernigan et al., 2017). Therefore, tillage could be exerting habitat filtering at the lower margin of the functional range by limiting the occurrence of species with low SLA, while not necessarily limiting the occurrence of species with more extreme values at the upper margin of the functional range, since high SLA values provide an adaptive advantage over tillage. Secondly, the habitat filtering could be hidden here by the abundance of species that are not typical of tillage (e.g. *Medicago minima*, *Bromus madritensis*) but which colonize these tilled spaces due to their massive occurrence in nearby mown plots, compatible with a spatial mass effect (Shmida and Wilson, 1985). Thus, the abundance in tilled non-irrigated plots of *M. minima and B. madritensis*, with LDMC values substantially higher than those of typical tillage species (e.g. *L. amplexicaule*), caused a marked increase in functional divergence (**Supplementary Figure 6D**).

The observed convergence in vegetative traits (PHV, LDMC, SLA) in tilled irrigated plots, would suggest a process of habitat filtering due to tillage. However, trait convergence was counterbalanced here by divergence patterns observed for certain reproductive traits (SM, SLI, OFL), which were markedly high for SM. Trait divergence for SLI and OFL was a consequence of the coexistence in these plots of tillage specialist species with a persistent seed bank and early flowering (e.g. *L. amplexicaule*, *Veronica hederifolia*) together with species with a transient seed bank (e.g. *Diplotaxis erucoides*, *M. minima*) and later flowering (e.g. *C. arvensis*, *B. madritensis*). This suggests the existence of a partitioning of phenological and regeneration niches (Grubb, 1977), with different reproductive strategies, similar to what has been documented in previous studies (Cornwell and Ackerly, 2009; Bernard‐Verdier et al., 2012). Likewise, the greatest functional divergence observed for SM was mainly due to the occurrence of a single species, *C. arvensis*, with a vastly higher SM than the others (**Figure 4B**). This higher SM may provide *C. arvensis* a greater ability to compete for establishment sites (Turnbull et al., 2004), increase success in colonization and survival after tillage or herbicide application (Kazakou et al., 2021) and establish a persistent soil seed bank with physically dormant seeds (Xiong et al., 2018), enabling its permanence on arable land.

Similarly, the higher abundance of *C. arvensis* in irrigated plots also led to a significantly higher functional divergence for SM compared to non-irrigated plots. This was also the cause of the higher divergence observed in irrigated plots for RLF, as the geophyte life form of *C. arvensis* contrasts with the vast majority of therophytes in these communities.

Overall, our results revealed that functional divergence was greater in irrigated than in non-irrigated plots, since beyond RLF and SM, trait divergence was detected in irrigated plots for PHV and LA. This is consistent with the literature, which states that functional divergence may increase with increasing water availability, particularly in vegetative traits related to resource acquisition (Spasojevic and Suding, 2012; Carmona et al., 2015; Nogueira et al., 2018). Divergence for PHV and LA was mainly driven by the increased abundance in irrigated plots of more competitive species such as *S. asper* or *D. erucoides* (Guerra et al., 2021), which had a significantly greater vegetative height and leaf size than the rest. The PHV and LA are closely associated with competitive ability (Grime, 1974; Keddy et al., 2002). On this basis, a tall and large-leaved species such as *S. asper* could compete more effectively for resources, especially for light capture and water. These trait divergence patterns observed for these vegetative traits would indicate a partitioning of light interception strategies, in line with previous work in herbaceous communities (Spasojevic and Suding, 2012).

From the above, irrigation favored more competitive species that showed markedly divergent values for certain traits. This led to an overdispersion of these traits and thus to an increase in functional divergence indices, as clearly reflected by the strong positive correlation found between ES RaoQ and C_index_ (**Figure 5**), albeit with a slight downward trend in functional divergence was observed at higher C_index_ values. This is consistent with former studies indicating that functional divergence may be maximal at intermediate levels of competition, whereas when competitive processes are maximized, as in undisturbed productive habitats, trait convergence may occur as weaker competitors are excluded and traits that confer plant species greater competitive ability are enhanced (Grime, 2006; Navas and Violle, 2009; Mayfield and Levine, 2010).

In our study, competitiveness was significantly greater in herbicide-treated irrigated plots, where a significant trait divergence was detected for the whole set of traits. The higher abundance of *S. asper* in these plots, where it is the dominant species together with *B. madritensis*, would explain the higher functional divergence observed. But to understand why, it is first necessary to describe the particular dynamics that occur in these herbicide-treated irrigated plots. Except for cases of herbicide resistance (Heap and Duke, 2018), which are not reported in our study field, the application of non-residual herbicide glyphosate removes almost the totality of weeds, thus resulting in a completely bare soil. But in our case, after the last summer application there was a time window between applications of 8-9 months (see **Figure 1**) during which different species were able to colonize, germinate and grow in gaps caused by the herbicide treatment. Under these particular conditions, the two dominant species were *S. asper* and *B. madritensis*, in our opinion due to two reasons: (a) the most abundant species in nearby plots might have colonized the herbicide-generated gaps more profusely. In fact, the two most abundant species in mown plots, *M. minima* and *B. madritensis*, were respectively the third and the first most abundant species in herbicide-treated irrigated plots (**Supplementary Figure 5**); (b) both tillage and mowing seem to be more successful in limiting the occurrence of *S. asper* (Guerra et al., 2022). During the months between the last herbicide application and the following year sampling, these dominant species initiate an annual process of resource competition which, as mentioned above, would be enhanced in irrigated plots. In this competition for resources, in line with the “limiting similarity” theory (Macarthur and Levins, 1967), two species are more likely to coexist if they have differentiated niches, as in the case of *S. asper* and *B. madritensis*, with markedly different functional traits (see **Supplementary Table 3**). Thus, with respect to vegetative traits, *S. asper* is a noticeably taller species, with much larger leaves, lower LDMC and lower SLA than *B. madritensis*. These species also differ in all reproductive traits, with *S. asper* having much lighter seeds, a markedly higher SLI, different phenological traits and a different dispersal syndrome than *B. madritensis*. Considering the evidence of two clearly divergent species within the multi-trait functional space, which was also reflected in significant trait divergence detected through null models, would confirm the existence of niche differentiation processes in herbicide-treated irrigated plots.

### 4.2 Synthesis and implications for agroecological weed management

Our study has evidenced how plant functional diversity can be affected by weed management through processes of trait convergence and divergence, also revealing that these processes can occur simultaneously, affecting both vegetative and reproductive plant traits. Trait divergence was significant in herbicide-treated irrigated plots, where marked differences between the two dominant species provided clear evidence of a process of niche differentiation. The effect of weed management on these processes was shaped by the application of irrigation in vineyard rows, leading primarily to functional divergence for vegetative traits linked to the ability to acquire resources.

On the whole, these community assembly processes were reflected in the estimated functional diversity indices. Hence, mown plots exhibited the highest functional richness. However, when species abundance was considered (i.e. RaoQ), herbicide-treated irrigated plots showed the highest functional diversity. Since ecosystem functions may be further conditioned by dominant plant species (mass-ratio hypothesis), RaoQ may be a more appropriate index to measure plant functional diversity. Indeed, higher functional diversity might not be a desirable indicator in agroecosystems, since higher RaoQ values were associated in this study with the occurrence of dominant species such as *S. asper* and *C. arvensis*, which are considered as noxious grapevine weeds (Guerra et al., 2022) and cause yield losses in a wide variety of crops (Boldt et al., 1998). Based on these assumptions, it would be advisable to propose agroecological weed management aimed not at maximizing functional diversity, but at favoring a certain range of plant trait values, reducing disservices (e.g. crop competition, pest hosting) and enhancing ecosystem services (e.g. pollination, carbon sequestration). For this purpose, CWM of plant traits could be a more useful tool than functional diversity indices (Lavorel, 2013; Weil et al., 2021). Therefore, further studies are required to clarify the relationship between weed management, plant functional traits and the provision of ecosystem services. This relationship should be the central core of agroecological weed management.

## Data availability statement

The original contributions presented in the study are included in the article/**Supplementary Material**. Further inquiries can be directed to the corresponding author.

## Author contributions

JG, FC, CF-Q and JD conceived and designed the research; JG and JD conducted the plant surveys; JG processed the data, obtained abundance matrix and collected data on plant traits; JG analysed the data with support from JP and JD; JG led the writing of the manuscript under the supervision of JD. All authors contributed critically to the drafts and gave final approval for publication.

## Funding

This work was supported by FEDER/MICINN-AEI, grant number AGL2017-83325-C4-1-R and by MICINN-AEI, grant number PID2020-113229RB-C41/AEI/10.13039/501100011033. The lead author has been granted a predoctoral research fellowship FPI-INIA2016-00035.

## Acknowledgments

We would like to thank the great support provided by J.M. Martín and D. Campos, CSIC technical staff who participated in the field sampling. We also acknowledge support of the publication fee by the CSIC Open Access Publication Support Initiative through its Unit of Information Resources for Research (URICI).

## Conflict of interest

The authors declare that the research was conducted in the absence of any commercial or financial relationships that could be construed as a potential conflict of interest.

## Publisher’s note

All claims expressed in this article are solely those of the authors and do not necessarily represent those of their affiliated organizations, or those of the publisher, the editors and the reviewers. Any product that may be evaluated in this article, or claim that may be made by its manufacturer, is not guaranteed or endorsed by the publisher.
