# Supplementary material for: Plant functional diversity is affected by weed management through processes of trait convergence and divergence
Source: Front Plant Sci. 2022 Oct 7;13:993051. doi: 10.3389/fpls.2022.993051 (PMC9585284; doi:10.3389/fpls.2022.993051)
Supplement: Supplementary file 1 [file DataSheet_1.pdf]

Supplementary Material

1 Functional diversity estimations

RaoQ index is strongly related to functional dispersion index (FDis) described by Laliberté and Legendre (2010) and to the community weighted variance (CWV) defined by Sonnier et al. (2010). FDis and CWV were initially calculated, yielding very similar results to RaoQ (Supplementary Figure 1). Consequently, RaoQ was used because it is a more common index than the previous ones in plant functional diversity studies.

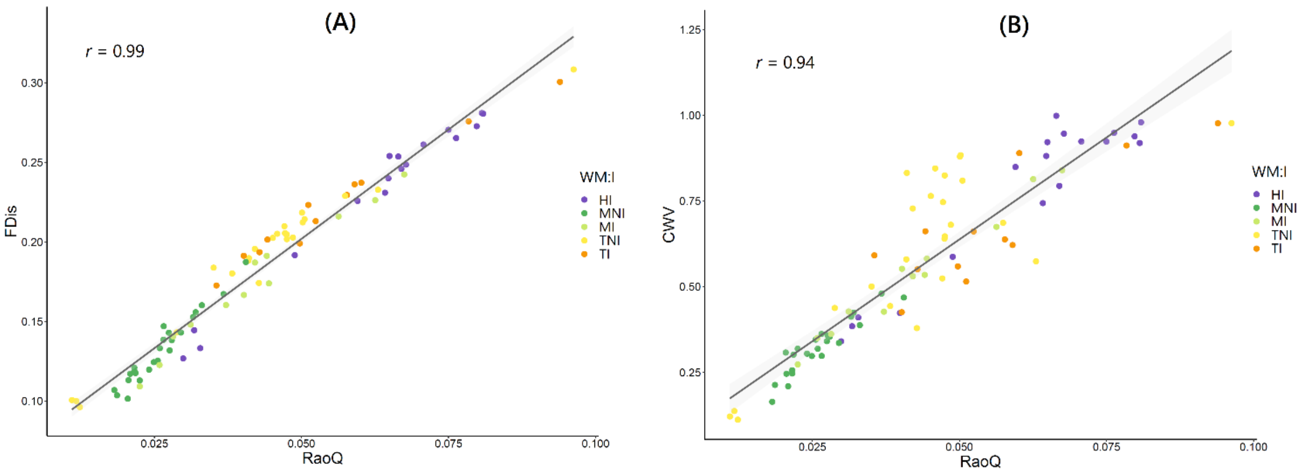

Supplementary Figure 1. Correlations between RaoQ and FDis (A) and RaoQ and CWV (B), measured through Spearman correlation test. Dots, corresponding to plots, have been filled in based on the interaction of weed management and irrigation (WM:I): HI, herbicide – irrigation; MNI, mowing – non-irrigation; MI, mowing – irrigation; TNI, tillage – non-irrigation; TI, tillage – irrigation.

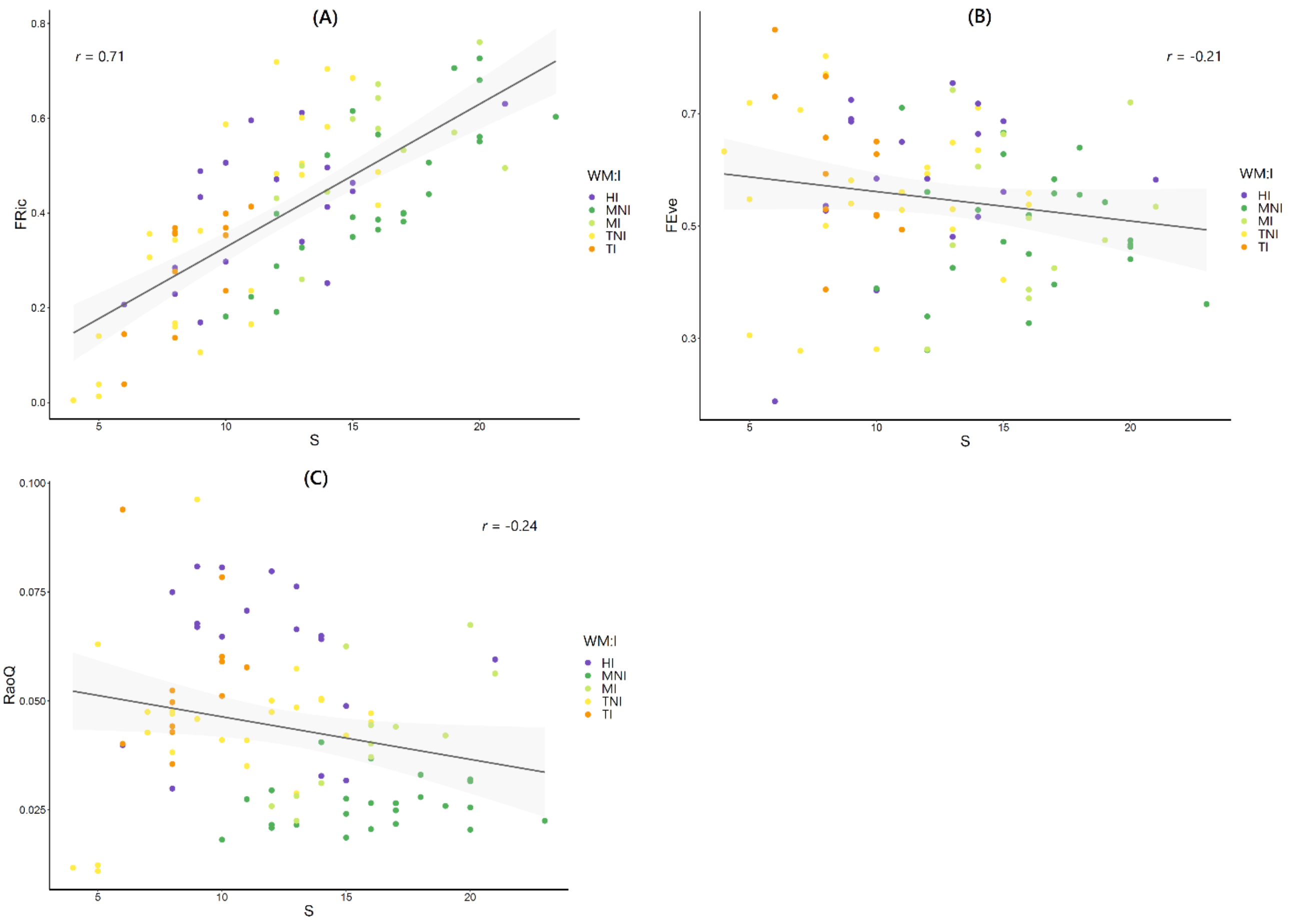

Supplementary Figure 2. Correlations between species richness (S) and functional richness (FRic), functional evenness (FEve) and Rao's quadratic entropy (RaoQ), measured through Spearman correlation test. Dots, corresponding to plots, have been filled in based on the interaction of weed management and irrigation (WM:I): HI, herbicide – irrigation; MNI, mowing – non-irrigation; MI, mowing – irrigation; TNI, tillage – non-irrigation; TI, tillage – irrigation.

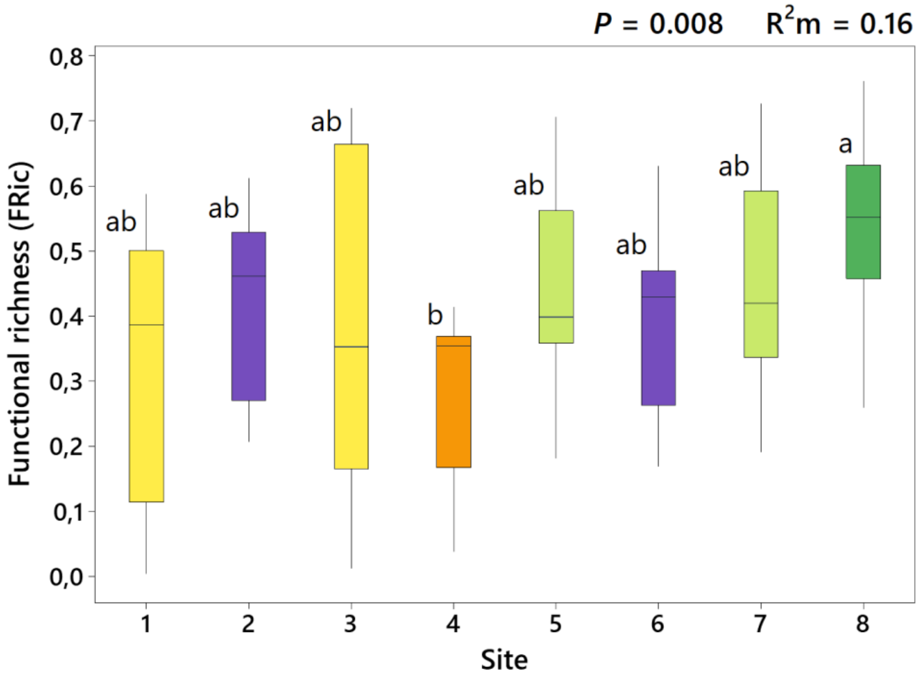

Supplementary Figure 3. Functional richness (FRic) according to site. Marginal *R*-squared (R^2m) indicate the variance explained by fixed factors. Same letters on the boxes indicate no significant differences. **Sites**: **1**, tilled inter-rows adjacent to herbicide-treated plots; **2**, herbicide-treated rows adjacent to tilled plots; **3**, tilled inter-rows adjacent to tilled plots; **4**, tilled rows; **5**, mown inter-rows adjacent to herbicide-treated plots; **6**, herbicide-treated rows adjacent to mown plots; **7**, mown inter-rows adjacent to mown plots; **8**, mown rows.

2 List of species and information on plant traits

Supplementary Table 1 List of plant species with a significant presence (relative cover > 0.25%) in the experimental field, ranked according to their relative cover

Species name	Family	Species code	Relative cover (%)
Medicago minima (L.) Bartal.	*Fabaceae*	**Med**	19.69
Bromus madritensis L.	*Poaceae*	**Bro**	14.48
Lamium amplexicaule L.	*Lamiaceae*	**Lam**	10.59
Convolvulus arvensis L.	*Convolvulaceae*	**Con**	6.57
Sonchus asper (L.) Hill	*Asteraceae*	**Son**	6.56
Veronica hederifolia L.	*Plantaginaceae*	**Ver**	5.00
Galium parisiense L.	*Rubiaceae*	**Gal**	4.37
Diplotaxis erucoides (L.) DC.	*Brassicaceae*	**Dip**	4.30
Crepis vesicaria subsp. *taraxacifolia* (Thuill.) Thell.	*Asteraceae*	**Cre**	3.76
Festuca myuros L.	*Poaceae*	**Vul**	3.06
Stellaria media (L.) Vill.	*Caryophyllaceae*	**Ste**	3.03
Astragalus hamosus L.	*Fabaceae*	**Ast**	2.86
Taraxacum obovatum (Willd.) DC.	*Asteraceae*	**Tar**	2.23
Fumaria officinalis L.	*Papaveraceae*	**Fum**	2.22
Cerastium glomeratum Thuill.	*Caryophyllaceae*	**Cer**	1.48
Papaver rhoeas L.	*Papaveraceae*	**Pap**	1.13
Malva sylvestris L.	*Malvaceae*	**Mal**	1.06
Hordeum murinum L.	*Poaceae*	**Hor**	0.84
Catapodium rigidum (L.) C.E.Hubb.	*Poaceae*	**Cat**	0.81
Festuca lachenalii (C.C.Gmel.) Spenn.	*Poaceae*	**Mic**	0.78
Geranium molle L.	*Geraniaceae*	**Ger**	0.75
Cirsium arvense (L.) Scop.	*Asteraceae*	**Cir**	0.71
Leontodon longirostris (Finch & P.D.Sell) Talavera	*Asteraceae*	**Leo**	0.61
Carduus bourgeanus Boiss. & Reut.	*Asteraceae*	**Car**	0.51
Scorzonera laciniata L.	*Asteraceae*	**Pod**	0.45
Lactuca serriola L.	*Asteraceae*	**Lac**	0.43
Erodium cicutarium (L.) L'Hér.	*Geraniaceae*	**Ero**	0.30
Rostraria cristata (L.) Tzvelev	*Poaceae*	**Ros**	0.28
Plantago lanceolata L.	*Plantaginaceae*	**Pla**	0.27

Supplementary Table 2 Relative cover of the most abundant species (relative cover > 3% on any of the five plot categories) according to WM:I

	HI	MNI	MI	TNI	TI
**Med**	0.13	0.39	0.33	0.10	0.05
**Bro**	0.28	0.10	0.13	0.12	0.14
**Lam**	0.02	0.00	0.00	0.24	0.23
**Con**	0.10	0.01	0.05	0.06	0.19
**Son**	0.21	0.01	0.08	0.01	0.04
**Ver**	0.01	0.01	0.01	0.13	0.08
**Gal**	0.04	0.09	0.08	0.01	0.00
**Dip**	0.05	0.01	0.02	0.06	0.12
**Cre**	0.04	0.10	0.06	0.00	-
**Vul**	0.01	0.07	0.06	0.00	0.00
**Ste**	0.00	0.00	0.00	0.10	0.02
**Ast**	0.02	0.07	0.04	0.00	-
**Tar**	0.01	0.04	0.05	0.00	0.01
**Fum**	0.00	-	-	0.05	0.05

HI, herbicide – irrigation; **MNI**, mowing – non-irrigation; **MI**, mowing – irrigation; **TNI**, tillage – non-irrigation; **TI**, tillage – irrigation

Supplementary Table 3 Trait data for the most abundant species (relative cover > 3% on any of the five plot categories). CSR coordinates are also indicated. Species have been ranked according to their relative cover. For further details about trait data, see FAWC Database in Guerra et al. (2021). Trait code: RLF, Raunkiær life form (G, geophyte; H, hemicryptophyte; Th, therophyte); PHV, plant height; LDMC, leaf dry matter content; LA, leaf area; SLA, specific leaf area; SM, seed mass; SLI, seedbank longevity index; DS, dispersal syndrome (U, unspecialized; W, anemochorous; Z, zoochorous); OFL, onset of flowering (e, earlier; m, medium; l, late); DFP, duration of flowering period.

Species	RLF	PHV (cm)	LDMC (mg · g^{-1})	LA (mm^2)	SLA (mm^2 · mg^{-1})	SM (g)	SLI	OFL	DFP	DS	C	S	R
**Med**	Th	13.93	269.69	91.34	22.72	1.07	0.21	m	4	Z	24	36	50
**Bro**	Th	21.51	263.30	132.80	26.50	2.64	0.35	m	3	Z	24	26	54
**Lam**	Th	17.04	140.85	182.77	28.39	0.56	0.60	e	5	U	24	8	70
**Con**	G	38.16	188.10	722.40	26.64	13.98	0.28	m	6	U	62	32	58
**Son**	Th	60.59	123.86	4279.35	19.97	0.28	0.73	l	6	W	60	4	36
**Ver**	Th	12.96	127.25	137.81	31.91	2.73	0.77	e	4	Z	24	0	66
**Gal**	Th	15.18	260.75	18.98	26.13	0.15	0.38	m	3	Z	24	26	64
**Dip**	Th	42.85	154.95	2296.00	20.69	0.21	0.00	e	8	Z	40	20	60
**Cre**	H	31.95	165.90	1024.32	18.99	0.41	0.00	m	3	W	50	38	26
**Vul**	Th	18.17	297.38	66.00	24.71	0.35	0.43	m	3	Z	24	34	52
**Ste**	Th	18.09	119.25	228.22	40.74	0.35	0.86	e	7	U	24	0	98
**Ast**	Th	15.22	235.73	56.06	19.13	2.81	0.50	m	3	U	24	42	46
**Tar**	H	7.50	136.98	3510.70	27.43	0.59	0.49	m	3	W	24	32	36
**Fum**	Th	26.37	166.97	526.00	27.58	3.26	0.63	e	6	U	24	14	64

3 Contribution of the most abundant species to trait divergence

The relationship between the most abundant species (relative cover > 3%) and ES RaoQ was explored by correlation test, considering data from all plots (Supplementary Figure 4) and plot data based on the interaction of weed management and position (Supplementary Figure 5). For data that fitted a normal distribution (ES RaoQ $_{LDMC}$) a Pearson correlation test was used. For the rest of the data, a Spearman correlation test was performed. Although both positive and negative correlations were represented using heatmaps, analysis focused on detecting positive correlations between relative species cover and ES RaoQ. Therefore, the value of moderate (0.4-0.6), strong (0.6-0.8) or very strong (0.8-1) positive correlations are indicated. These correlations must be analyzed with caution, as the correlation between a species and ES RaoQ can be positive or negative depending on the plots considered. For instance, *B. madritensis* showed a positive correlation with ES RaoQ $_{LDMC}$ in tilled rows, where its LDMC (263.30) differs with the community weighted mean (CWM) for these plots (177.95); but exhibited a negative correlation in mown inter-rows, where its LDMC is close to the CWM (241.33). Furthermore, it should be noted that the fact that there might be a positive correlation between one variable and another does not necessarily imply that there exists functional divergence, but rather that species contribute to more divergent values. In addition, bubble plots were employed to display the position of the most abundant species along the functional space for six quantitative traits (Supplementary Figure 6).

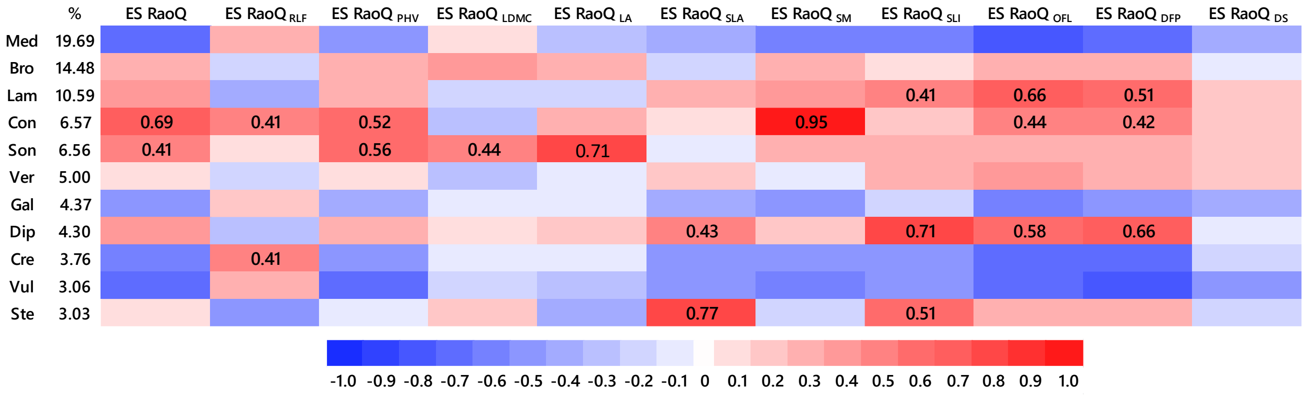

Supplementary Figure 4. Correlation heatmap (considering all plots) displaying the results of correlation test between the most abundant species and the RaoQ effect size (ES RaoQ) calculated for the set of all traits and for each single trait. Species have been ranked according to their relative cover (%). Species codes are indicated in Supplementary Table 1.

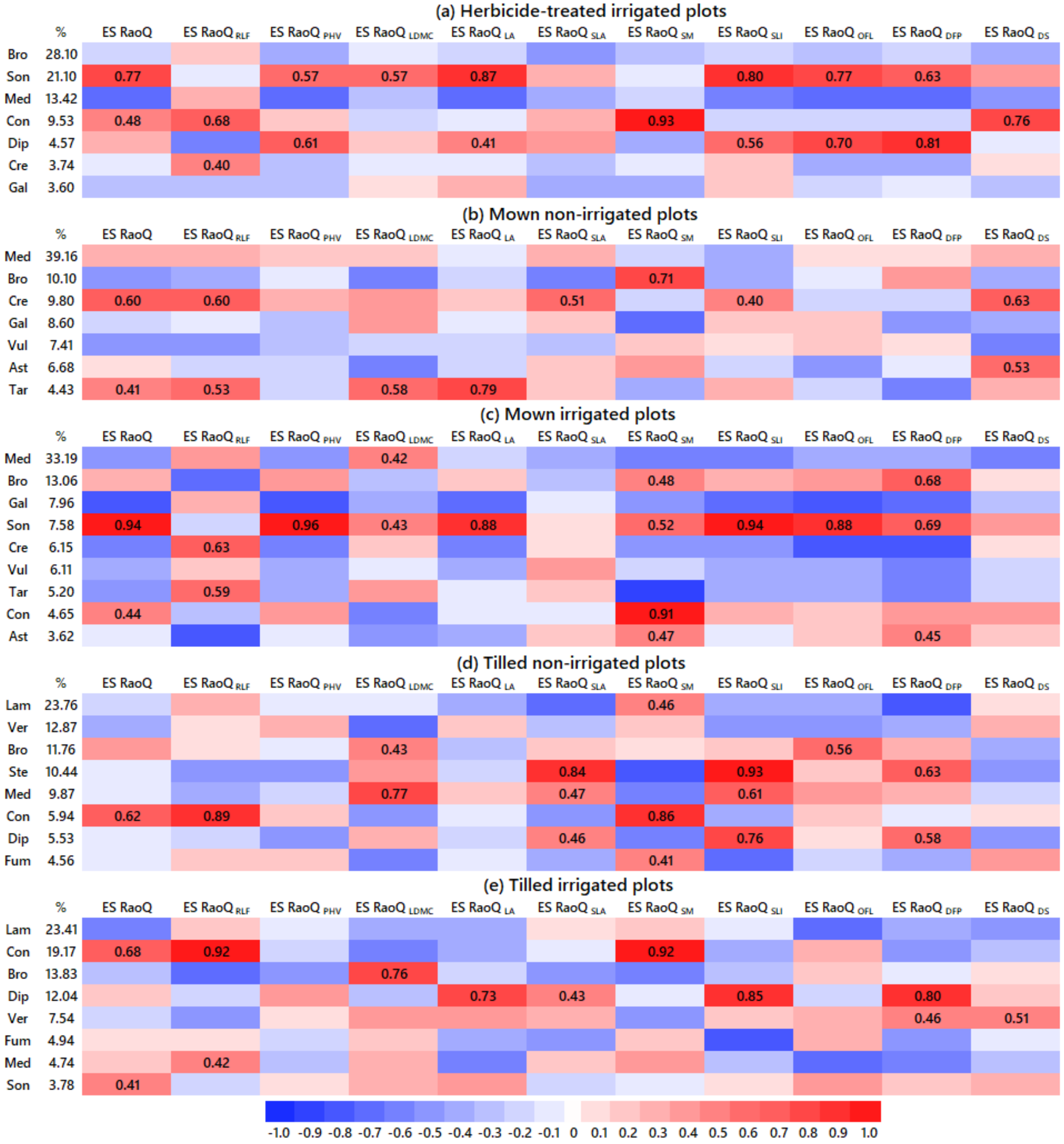

Supplementary Figure 5. Correlation heatmaps displaying the results of correlation test between the most abundant species and the RaoQ effect size (ES RaoQ) calculated for the set of all traits and for each single trait. Correlations were calculated from plot data based on the interaction of weed management and position. Species have been ranked according to their relative cover (%). Species codes are indicated in Supplementary Table 1.

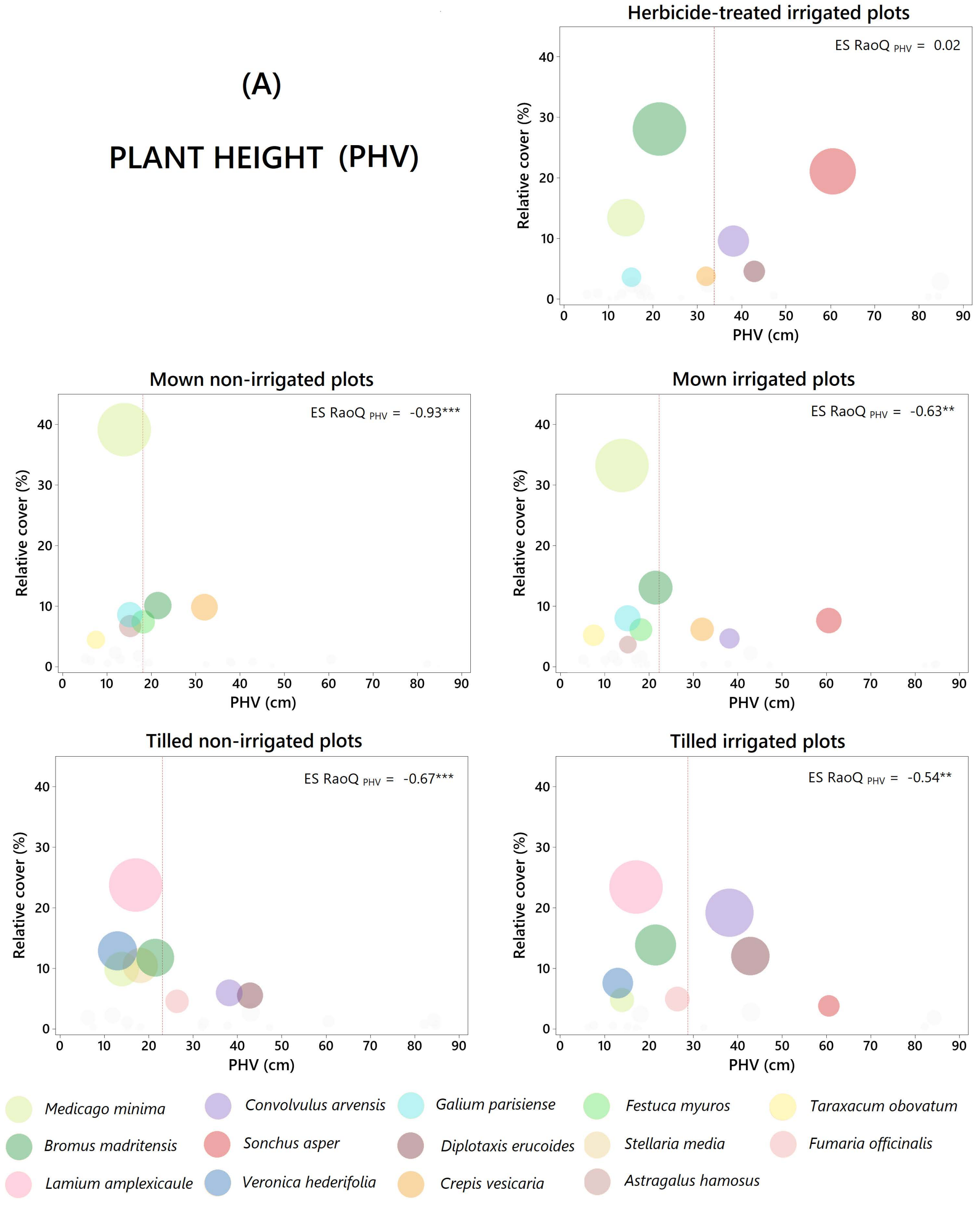
(A)
PLANT HEIGHT (PHV)
Herbicide-treated irrigated plots
ES RaoQ PHV = 0.02
Mown non-irrigated plots
ES RaoQ PHV = -0.93***
Mown irrigated plots
ES RaoQ PHV = -0.63**
Tilled non-irrigated plots
ES RaoQ PHV = -0.67***
Tilled irrigated plots
ES RaoQ PHV = -0.54**
Relative cover (%)
PHV (cm)
Medicago minima
Convolvulus arvensis
Galium parisiense
Festuca myuros
Taraxacum obovatum
Bromus madritensis
Sonchus asper
Diplotaxis erucoides
Stellaria media
Fumaria officinalis
Lamium amplexicaule
Veronica hederifolia
Crepis vesicaria
Astragalus hamosus

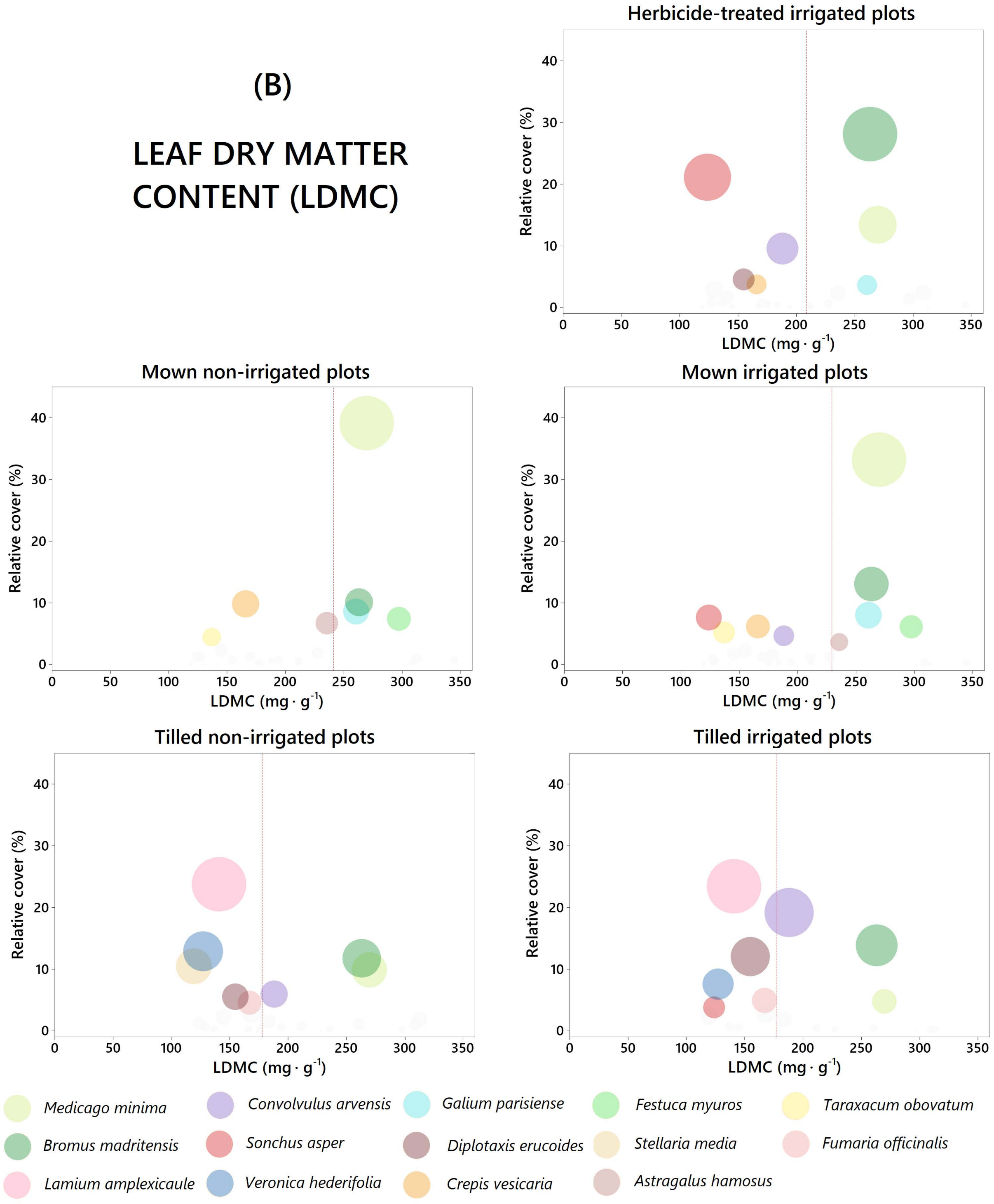
(B)
LEAF DRY MATTER CONTENT (LDMC)
Herbicide-treated irrigated plots
Mown non-irrigated plots
Mown irrigated plots
Tilled non-irrigated plots
Tilled irrigated plots
Relative cover (%)
LDMC (mg · g-1)
Medicago minima
Convolvulus arvensis
Galium parisiense
Festuca myuros
Taraxacum obovatum
Bromus madritensis
Sonchus asper
Diplotaxis erucoides
Stellaria media
Fumaria officinalis
Lamium amplexicaule
Veronica hederifolia
Crepis vesicaria
Astragalus hamosus

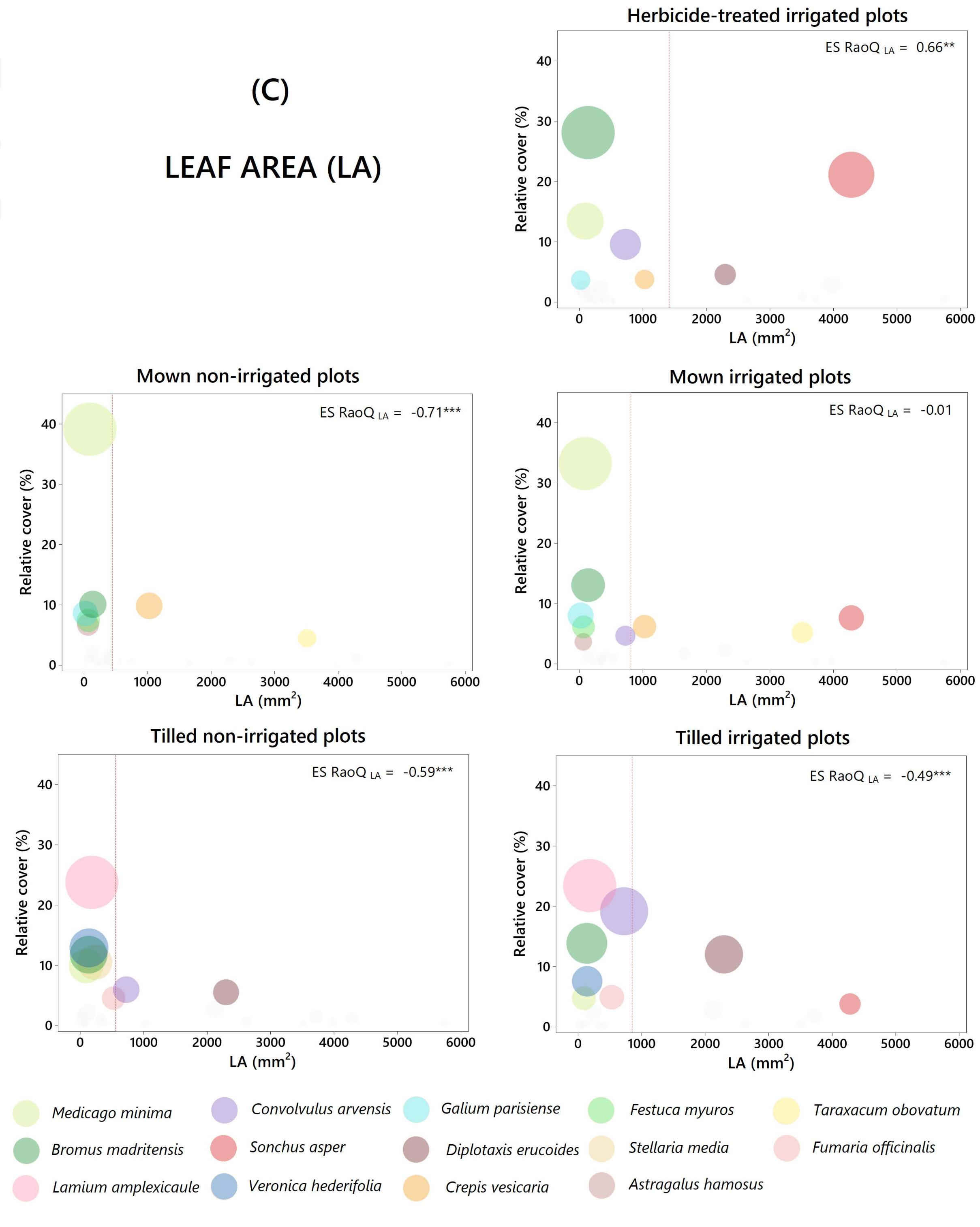
(C)
LEAF AREA (LA)
Herbicide-treated irrigated plots
ES RaoQ LA = 0.66**
Mown non-irrigated plots
ES RaoQ LA = -0.71***
Mown irrigated plots
ES RaoQ LA = -0.01
Tilled non-irrigated plots
ES RaoQ LA = -0.59***
Tilled irrigated plots
ES RaoQ LA = -0.49***
Relative cover (%)
LA (mm²)
0
10
20
30
40
1000
2000
3000
4000
5000
6000
Medicago minima
Convolvulus arvensis
Galium parisiense
Festuca myuros
Taraxacum obovatum
Bromus madritensis
Sonchus asper
Diplotaxis erucoides
Stellaria media
Fumaria officinalis
Lamium amplexicaule
Veronica hederifolia
Crepis vesicaria
Astragalus hamosus

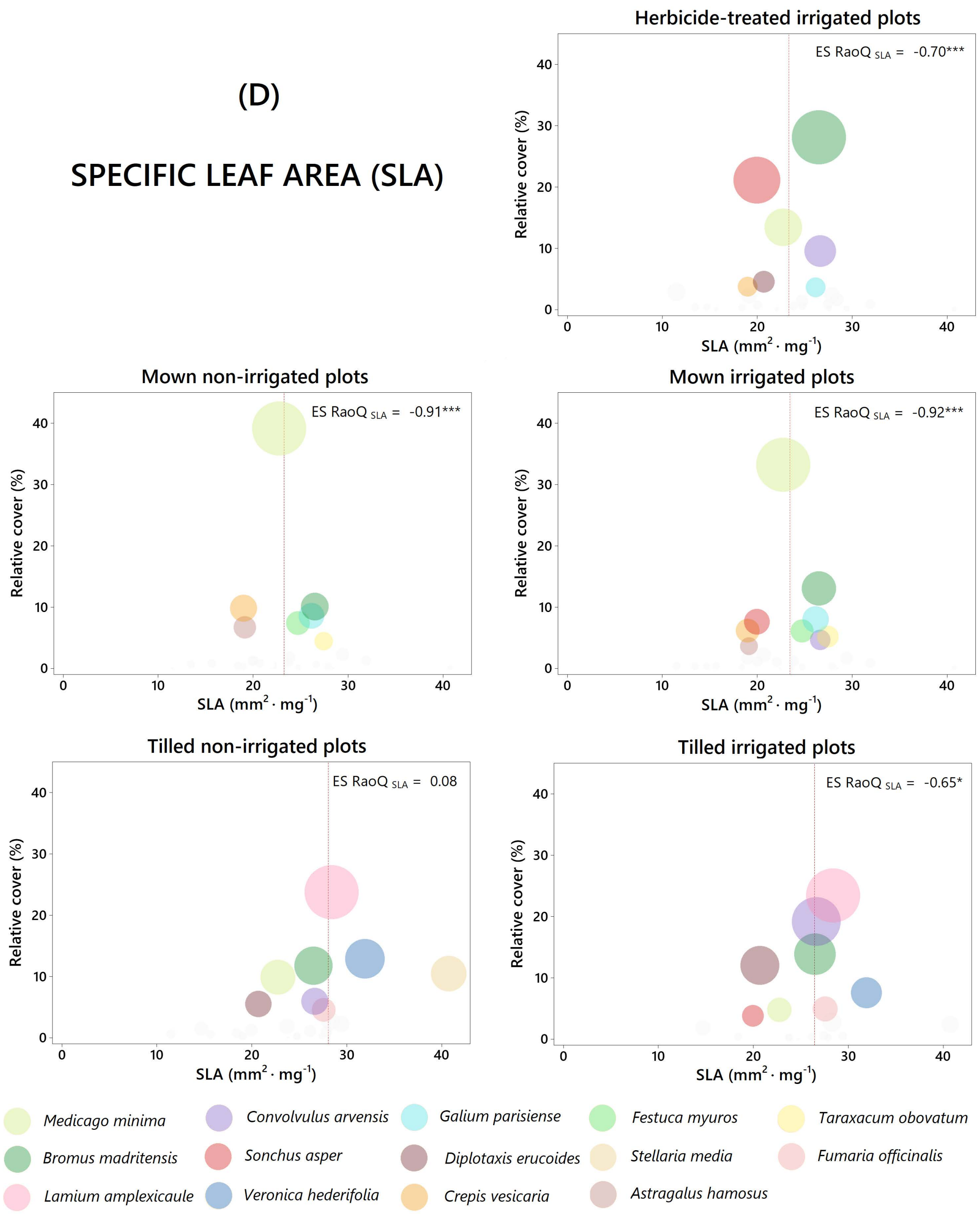
(D)
SPECIFIC LEAF AREA (SLA)
Herbicide-treated irrigated plots
ES RaoQ SLA = -0.70***
Mown non-irrigated plots
ES RaoQ SLA = -0.91***
Mown irrigated plots
ES RaoQ SLA = -0.92***
Tilled non-irrigated plots
ES RaoQ SLA = 0.08
Tilled irrigated plots
ES RaoQ SLA = -0.65*
Relative cover (%)
SLA (mm2 · mg-1)
0
10
20
30
40
Medicago minima
Convolvulus arvensis
Galium parisiense
Festuca myuros
Taraxacum obovatum
Bromus madritensis
Sonchus asper
Diplotaxis erucoides
Stellaria media
Fumaria officinalis
Lamium amplexicaule
Veronica hederifolia
Crepis vesicaria
Astragalus hamosus

(E)

SEED MASS (SM)

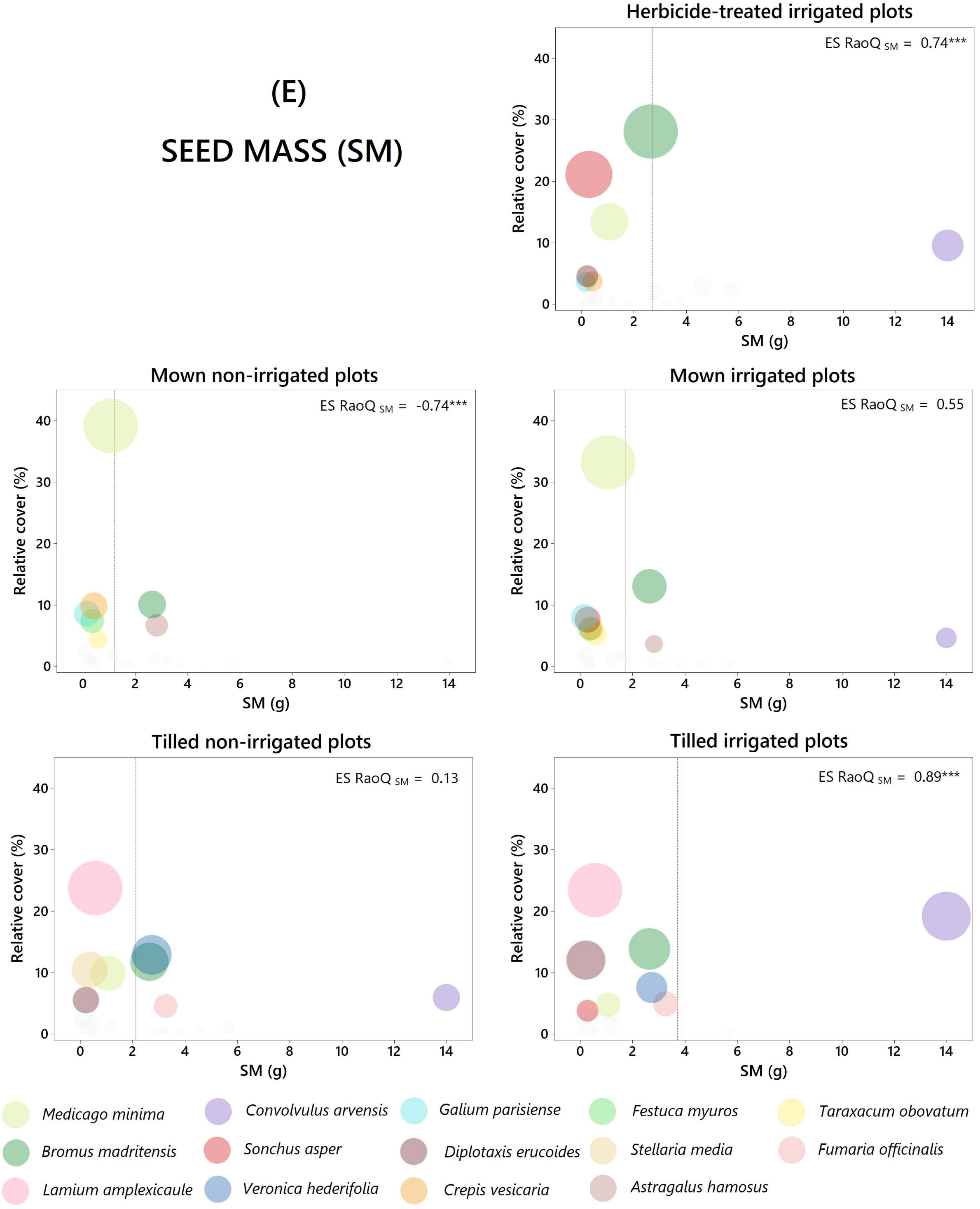

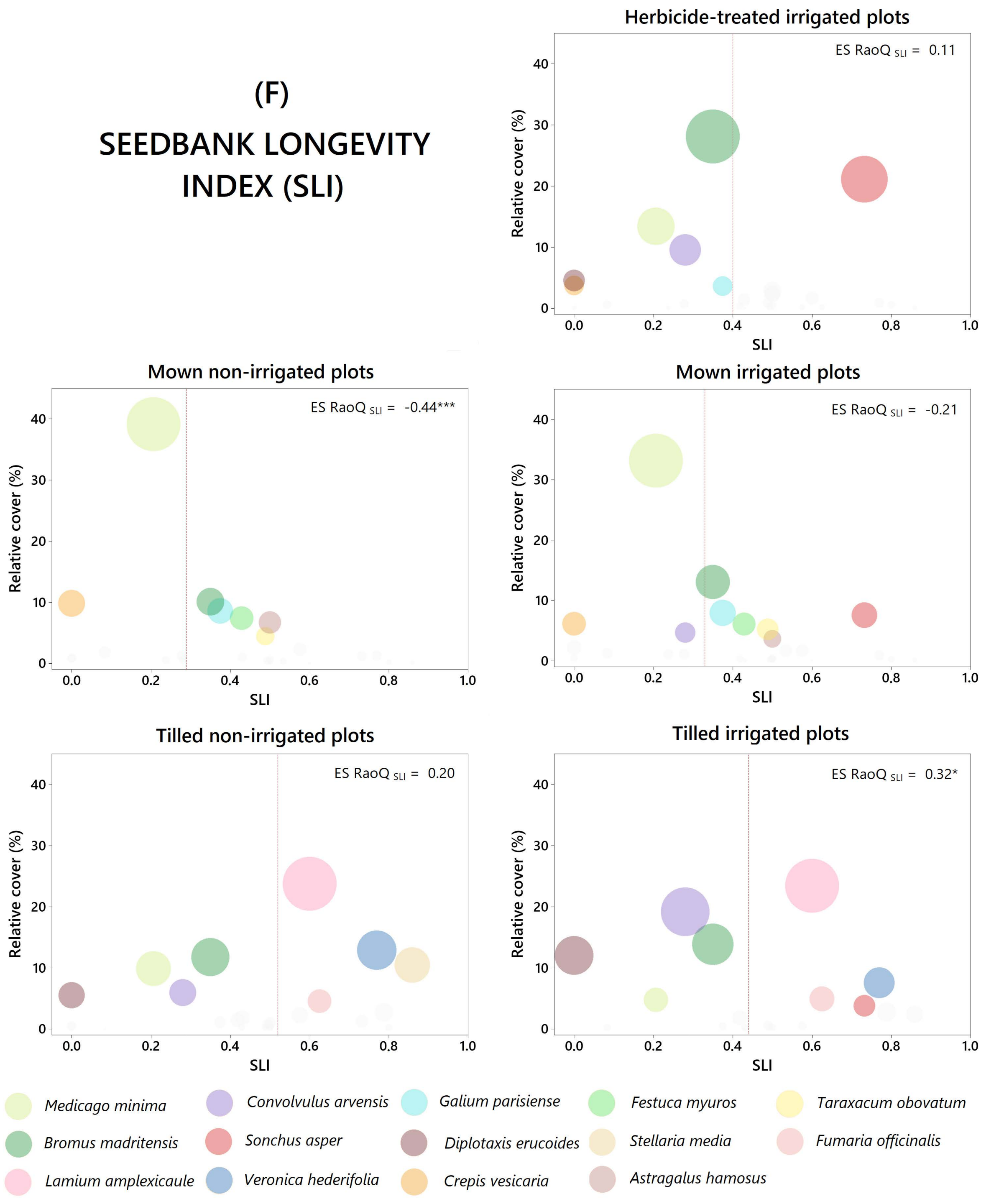

Supplementary Figure 6. Bubble plots displaying the position of the most abundant species within the functional space on six quantitative traits: (A) plant height, (B) leaf dry matter content, (C) leaf area, (D) specific leaf area, (E) seed mass and (F) seedbank longevity index. Vertical red lines indicate the CWM of each trait in each plot type according to Guerra et al. (2021). Bubble size is proportional to the relative abundance of plant species.

4 Exploratory analyses of individual effects of fixed factors

Supplementary Table 4. Exploratory analysis of the effect of weed management (H, herbicide; M, mowing; T, tillage) on trait convergence and divergence. To examine differences due to weed management, only data from vineyard rows were used. For models built for ES RaoQ the variance explained by weed management (R^2) and the level of significance (*P< 0.05, **P< 0.01, ***P< 0.001) are indicated in italics. For each management the mean ES RaoQ values are given. Means with different letters in the same column are significantly different.

	**ES RaoQ $_{RLF}$**	**ES RaoQ $_{PHV}$**	**ES RaoQ $_{LDMC}$**	**ES RaoQ $_{LA}$**	**ES RaoQ $_{SLA}$**	**ES RaoQ $_{SM}$**	**ES RaoQ $_{SLI}$**	**ES RaoQ $_{OFL}$**	**ES RaoQ $_{DFP}$**	**ES RaoQ $_{DS}$**
R^2	*0,03*	***0.27****	***0.26****	***0.33****	***0.22***	***0.21***	***0.19***	***0.38****	***0.29****	***0.28****
**H**	-0.08 a	-0.05 a	0.16 a	0.41 a	- 0.55 a	0.65 ab	0.01 ab	0.01 a	0.14 a	0.04 a
**M**	0.09 a	-0.53 b	-0.11 ab	-0.08 b	-0.92 b	0.34 b	-0.25 b	-0.68 b	-0.43 b	-0.66 b
**T**	0.02 a	-0.43 b	-0.37 b	-0.40 b	- 0.41 a	0.81 a	0.29 a	0.36 a	0.14 a	-0.34 ab

Trait code indicated in the subscripts: **RLF**, Raunkiær Life Form; **PHV**, plant height; **LDMC**, leaf dry matter content; **LA**, leaf area; **SLA**, specific leaf area; **SM**, seed mass; **SLI**, seedbank longevity index; **DS**, dispersal syndrome; **OFL**, onset of flowering; **DFP**, duration of flowering period

Supplementary Table 5. Exploratory analysis of the effect of irrigation (NI, non-irrigation; I, irrigation) on trait convergence and divergence. Herbicide-treated plots were excluded as the herbicide treatment was carried out only on rows. For models built for ES RaoQ the variance explained by position (R^2) and the level of significance (*P<0.05, **P < 0.01, ***P < 0.001) are indicated in italics. For each position the mean ES RaoQ values are indicated. Means with different letters in the same column are significantly different.

	**ES RaoQ $_{RLF}$**	**ES RaoQ $_{PHV}$**	**ES RaoQ $_{LDMC}$**	**ES RaoQ $_{LA}$**	**ES RaoQ $_{SLA}$**	**ES RaoQ $_{SM}$**	**ES RaoQ $_{SLI}$**	**ES RaoQ $_{OFL}$**	**ES RaoQ $_{DFP}$**	**ES RaoQ $_{DS}$**
R^2	***0.11****	***0.10***	*0,01*	***0.19****	*0,03*	***0.27****	*0,03*	*0,03*	*0,04*	*0.00*
**NI**	-0.29 b	-0.72 b	-0.17 a	-0.61 b	-0.45 a	-0.15 b	-0.15 a	-0.40 a	-0.38 a	-0.41 a
**I**	0.06 a	-0.48 a	0.07 a	-0.24 a	-0.67 a	0.57 a	0.02 a	-0.16 a	-0.14 a	-0.50 a

Trait code indicated in the subscripts: **RLF**, Raunkiær Life Form; **PHV**, plant height; **LDMC**, leaf dry matter content; **LA**, leaf area; **SLA**, specific leaf area; **SM**, seed mass; **SLI**, seedbank longevity index; **DS**, dispersal syndrome; **OFL**, onset of flowering; **DFP**, duration of flowering period.

5 Exploratory analyses of individual effects of fixed factors

Supplementary Table 6. Results of Spearman's correlation test between the competitiveness index (C_{index}) and the RaoQ effect size (ES RaoQ) calculated for the set of all traits and for each single trait.

	ES RaoQ	ES RaoQ $_{RLF}$	ES RaoQ $_{PHV}$	ES RaoQ $_{LDMC}$	ES RaoQ $_{LA}$	ES RaoQ $_{SLA}$	ES RaoQ $_{SM}$	ES RaoQ $_{SLI}$	ES RaoQ $_{OFL}$	ES RaoQ $_{DFP}$	ES RaoQ $_{DS}$
C_{index}	**0.80*** **	**0.39*** **	**0.74*** **	0,08	**0.60*** **	0,17	**0.73*** **	**0.42*** **	**0.51*** **	**0.48*** **	**0.30** **

Trait code indicated in the subscripts: **RLF**, Raunkiær Life Form; **PHV**, plant height vegetative; **LDMC**, leaf dry matter content; **LA**, leaf area; **SLA**, specific leaf area; **SM**, seed mass; **SLI**, seedbank longevity index; **OFL**, onset of flowering; **DFP**, duration of flowering period; **DS**, dispersal syndrome
